# General population-based lung function trajectories over the life course: an accelerated cohort study

**DOI:** 10.1016/S2213-2600(25)00043-8

**Published:** 2025-07

**Authors:** Judith Garcia-Aymerich, Martí de las Heras, Anne-Elie Carsin, Simone Accordini, Alvar Agustí, Dinh Bui, Shyamali C Dharmage, James W Dodd, Ikenna Eze, Ulrike Gehring, Thorarinn Gislason, Raquel Granell, Medea Imboden, Carmen Íñiguez, Ayoung Jeong, Sarah Koch, Gerard H Koppelman, Bénédicte Leynaert, Erik Melén, Jennifer Perret, Nicole Probst-Hensch, Loreto Santa-Marina, Maties Torrent, Don Vicendese, Judith M Vonk, Maaike de Vries, E Haydn Walters, Gang Wang, Jadwiga A Wedzicha, Deborah Jarvis, Rosa Faner

**Affiliations:** a ISGlobal, Barcelona, Spain; b Universitat Pompeu Fabra, Barcelona, Spain; c CIBER Epidemiología y Salud Pública, Madrid, Spain; d Unit of Epidemiology and Medical Statistics, Department of Diagnostics and Public Health, University of Verona, Istituti Biologici II, Verona, Italy; e Medicine Department, University of Barcelona, Barcelona, Spain; f Biomedicine Department, University of Barcelona, Barcelona, Spain; g Respiratory Institute, Clinic Barcelona, Barcelona, Spain; h FCRB-IDIBAPS, Barcelona, Spain; i CIBERES, Madrid, Spain; j Allergy and Lung Health Unit, School of Population and Global Health, University of Melbourne, Melbourne, VIC, Australia; k Academic Respiratory Unit, NIHR Bristol Biomedical Research Centre and Bristol Medical School, University of Bristol, Bristol, UK; l Swiss Tropical and Public Health Institute, Allschwil, Switzerland; m University of Basel, Basel, Switzerland; n Institute for Risk Assessment Sciences, Utrecht University, Utrecht, Netherlands; o Department of Sleep, Landspitali University Hospital, Reykjavik, Iceland; p Faculty of Medicine, University of Iceland, Reykjavik, Iceland; q MRC Integrative Epidemiology Unit, Department of Population Health Sciences, Bristol Medical School, University of Bristol, Bristol, UK; r Department of Statistics and Operational Research, University of Valencia, Burjassot, Spain; s Department of Pediatric Pulmonology and Pediatric Allergology, Beatrix Children's Hospital, University Medical Center Groningen, University of Groningen, Groningen, Netherlands; t GRIAC Research Institute, University Medical Center Groningen, University of Groningen, Groningen, Netherlands; u Department of Epidemiology, University Medical Center Groningen, University of Groningen, Groningen, Netherlands; v Université Paris-Saclay, UVSQ, Université Paris-Sud, Inserm, CESP, Équipe d'Épidémiologie Respiratoire Intégrative, Villejuif, France; w Department of Clinical Science and Education Södersjukhuset, Karolinska Institutet, Stockholm, Sweden; x Group of Environmental Epidemiology and Child Development, Biogipuzkoa Health Research Institute, San Sebastian, Spain; y Department of Health of the Basque Government, San Sebastian, Spain; z IB-SALUT, Àrea de Salut de Menorca, IdISBa, Maó, Spain; aa School of Computing, Engineering and Mathematical Sciences, La Trobe University, Melbourne, VIC, Australia; ab Division of Internal Medicine, Institute of Integrated Traditional Chinese and Western Medicine, West China Hospital, Sichuan University, Chengdu, China; ac National Heart and Lung Institute, Imperial College London, London, UK

## Abstract

**Background:**

Lung function is a key determinant of health, but current knowledge on lung function growth and decline over the life course is based on fragmented, potentially biased data. We aimed to empirically derive general population-based life course lung function trajectories, and to identify breakpoints and plateaus.

**Methods:**

We created an accelerated cohort by pooling data from eight general population-based child and adult cohort studies from Europe and Australia. We included all participants with information on lung function, smoking status, BMI, and asthma diagnosis status from at least two visits. We used cross-classified three-level linear mixed models to derive sex-specific life course trajectories of FEV_1_, forced vital capacity (FVC), and FEV_1_/FVC ratio based on observations at ages 4–80 years, and Bayesian time-series decomposition to identify breakpoints and plateaus. We repeated sex-specific analyses with separate stratification for asthma status (never had asthma *vs* persistent asthma, where persistent was defined as the risk factor being present at all participant visits) and smoking status (never smoker *vs* persistent smoker).

**Findings:**

The accelerated cohort included 30 438 participants born between 1901 and 2006 (15 703 [51·6%] female and 14 735 [48·4%] male; mean age 26 [SD 16] years), who provided a total of 87 666 observations (range 2–8 observations per participant). In female participants, FEV_1_ increased non-linearly in two phases, at a mean of 234 (95% CI 223 to 245) mL/year until age 13 (95% credible interval [CrI] 12 to 15) years, then at 99 (76 to 122) mL/year until a peak at age 20 (18 to 22) years, and subsequently decreased throughout the rest of adulthood (−26 [−27 to −25] mL/year). In male participants, the pattern was similar, with an increase in FEV_1_ of 271 (263 to 280) mL/year until age 16 (14 to 18) years, which slowed to 108 (93 to 124) mL/year until reaching a maximum at age 23 (21 to 25) years, decreasing thereafter (−38 [−39 to −37] mL/year), representing a later peak than in female participants. In female participants, FVC increased non-linearly in two phases, at 232 (95% CI 222 to 243) mL/year until age 14 (95% CrI 12 to 15) years, then at 77 (59 to 94) mL/year until peaking at age 20 (19 to 22) years, after which it decreased throughout the rest of adulthood (−26 [−27 to −25] mL/year). In male participants, FVC also increased in two phases, at 326 (315 to 337) mL/year until age 15 (13 to 17) years, then at 156 (144 to 168) mL/year until a peak at 23 (19 to 30) years, and subsequently declined in two phases (−22 [−29 to −14] mL/year until age 42 [38 to 50] years, then −36 [−38 to −34] mL/year thereafter). No plateau after the peak was observed for either lung function parameter in both sexes. FEV_1_/FVC ratio decreased throughout life from the starting age of 4 years in both sexes with some distinct patterns. Stratified analysis showed that persistent asthma (*vs* never had asthma) was related to an earlier FEV_1_ peak, lower FEV_1_ throughout adulthood, and lower FEV_1_/FVC ratio across the life course in both sexes. Persistent smoking (*vs* never smoking) was related to an accelerated decrease in FEV_1_ and FEV_1_/FVC ratio during adulthood in both sexes. No statistically significant plateau was observed in any lung function parameter across the strata of asthma or smoking status.

**Interpretation:**

In both sexes, FEV_1_ and FVC increased in two phases, with a fast increase until around age 13–16 years, and then a slower increase until a peak. Neither parameter showed a plateau phase after the peak, and decreases started earlier than previously described. FEV_1_/FVC ratio decreased throughout life. These observations provide an essential, but previously unavailable, framework to assess and monitor lung health over the life course.

**Funding:**

EU Horizon 2020, Wellcome, European Respiratory Society, AstraZeneca, Chiesi, GSK, Menarini Group, and Sanofi.

## Introduction

Lung function is a key determinant of health throughout the lifespan. Current understanding of the normal lung function trajectory over the lifetime postulates the existence of different phases: a slow increase during childhood (lag phase), a rapid acceleration during puberty, the attainment of a peak at around age 20–25 years (earlier in women than men), a plateau phase after the peak that has been proposed to last up to 20 years in adulthood and, finally, a phase of decline due to physiological lung ageing.^1^

**Research in context**

**Evidence before this study**

We searched PubMed for articles in any language published from database inception to Oct 12, 2024, with the terms: (“lung function”[tiab] OR “pulmonary function”[tiab] OR “FEV1”[tiab] OR “FVC”[tiab]) AND (curv*[tiab] OR trajector*[tiab] OR plateau[tiab] OR breakpoint[tiab] OR rate[tiab] OR growth[tiab] OR decline[tiab]) AND (“life course”[tiab] OR lifecourse[tiab] OR cohort[tiab] OR follow-up[tiab]) AND (population-based[tiab]). From 258 results, we excluded one abstract book on intensive care and emergency medicine, five study protocols, two narrative reviews (providing no lung function data), 11 articles without lung function data, and 71 papers focused on individuals with various chronic health conditions. Of the remaining 168 studies, 95 reported lung function measurements at a single timepoint or their determinants or outcomes either in children or adults (without providing rates of increase or decrease), six focused on rates, determinants, or outcomes of lung function growth in children or adolescents (excluding adults), 57 focused on rates, determinants, or outcomes of lung function decline in adults (excluding children), and ten described different lung function trajectory groups but did not report rates. No study examined rates of lung function growth and decline across the full life course. Evidence was scarce on the shape of lung function growth in infancy and adolescence, the ages at which lung function peaks and decline onset occurs, and whether a plateau phase occurs.

**Added value of this study**

To our knowledge, this study is the first to derive general population-level lung function trajectories from childhood to late adulthood, using an accelerated cohort design. The key findings were that, firstly, increases in FEV_1_ and forced vital capacity (FVC) during childhood and adolescence were not linear but occurred in two phases, with a fast increase beginning in early childhood (in this analysis, measured from age 4 years), followed by a slower increase from age 13–16 years until a peak. Contrary to previous assumptions, we observed no lag period, meaning no phase of little to no increase in the parameters, between ages 4 and 10 years. Secondly, FEV_1_ and FVC peaked earlier in female participants (at around age 20 years for both parameters, which is markedly later than in most previous reports) than in male participants (who had peaks at around age 23 years for both FEV_1_ and FVC). Thirdly, we found no evidence of a plateau after the FEV_1_ or FVC peaks, not even among people who had never had asthma or never smoked, unlike some previous studies and models that have suggested a plateau up to age 40 years. Fourthly, decreases in FEV_1_ and FVC began immediately after the peaks in the mid-20s, much earlier than previously described, with the decrease ranging from 22 to 38 mL/year. And finally, FEV_1_/FVC ratio decreased throughout life, at a varying rate depending on age and sex.

**Implications of all the available evidence**

This study underscores the use of spirometry early in life to identify individuals at risk of adult chronic respiratory disease and emphasises the need to promote respiratory health from early childhood through to early adulthood to support lung growth and prevent low peak lung function. Our findings provide a crucial yet previously unavailable framework for assessing and monitoring lung health across the life course. Additionally, they provide strong support for approaches to re-analyse existing health megadata to establish more precise reference data and eventually test how and when early-life factors affect health and disease later in life.

This model originates from either cross-sectional or longitudinal studies that do not cover the full life course, with both approaches potentially resulting in biased estimates due to regression to the mean, or birth cohort and survivor effects.^2^, ^3^, ^4^, ^5^, ^6^ Furthermore, uncertainties remain about whether the increase in lung function in infancy and adolescence is linear or not, the ages at which peak function and onset of decline occur, whether a plateau phase really occurs, and what the normal slope is of lung function decline.^1^, ^7^

To address these gaps, the ideal study design should include a general population-based sample of healthy individuals born in different years^8^ and followed up from birth to death. Such a study is missing and is unlikely to be performed considering the time and cost required. However, re-analysing existing lung function data from multiple general population-based studies using alternative analytical methods to mimic such a life-long cohort might be an efficient way to use available resources and fill the knowledge gaps.^7^, ^9^ One such method is the accelerated cohort design, in which data from multiple established cohort studies, starting at different ages, are pooled to cover the desired age range.^10^ This approach has successfully been applied to investigate age-related health parameters such as blood pressure and psychiatric traits^11^, ^12^ but, to date, it has never been used to investigate lung function trajectories during the life course.

We hypothesised that an accelerated cohort design that pooled several large existing general population-based cohorts could be used to empirically develop lung function trajectories over the lifespan in the general population. In this study, by pooling eight general population-based child and adult cohort studies using an accelerated cohort design, we sought to: (1) derive sex-specific, general population-level trajectories of the three main forced spirometry parameters (FEV_1_, forced vital capacity [FVC], and their ratio [FEV_1_/FVC]) over the life course; and (2) identify the ages at which these derived trajectories exhibit breakpoints (ie, points at which trajectory changes occur), estimate the magnitude of increase or decrease in the segments defined between these breakpoints, and investigate the potential presence of plateau phases. Collectively, this new information could provide a reference framework for the interpretation of lung function measures and changes throughout life, by improving the identification of deviation from average, and subsequently allow the instigation of appropriate prevention and treatment, with the long-term aim of promoting human health and healthier ageing. Specifically, for individuals, it might offer insights into their lung function trajectory and its associated health implications; for clinicians, it might support preventive and therapeutic decision making, potentially in combination with tools such as the recently developed Lung Function Tracker;^13^ for policymakers, it might aid in timing public health initiatives focused on preventing and diagnosing respiratory diseases early in life; and for the pharmaceutical industry, it might facilitate assessment of the effects of drugs on lung function. Together, such applications could help to improve respiratory health outcomes across populations.

## Methods

### Study design and participants

As part of the Chronic Airway Diseases Early Stratification European Respiratory Society Clinical Research Collaboration,^14^ we created an accelerated cohort^10^ by pooling participants from eight general population-based child and adult prospective studies from Europe and Australia, covering individuals aged 4–82 years: the Avon Longitudinal Study of Parents and Children (known as ALSPAC) in the UK;^15^ the Children, Allergy, Milieu, Stockholm, Epidemiology (BAMSE) study in Sweden;^16^ the European Community Respiratory Health Survey (ECRHS) in Belgium, Denmark, France, Germany, Iceland, Italy, Norway, Spain, Sweden, Switzerland, and the UK in Europe, and in Australia;^17^ the Infancia y Medio Ambiente (INMA) study birth cohorts from Sabadell, Valencia, Menorca, and Gipuzkoa, Spain;^18^ the Prevention and Incidence of Asthma and Mite Allergy (PIAMA) study in the Netherlands;^19^ the Swiss Study on Air Pollution and Lung Disease in Adults (SAPALDIA) in Switzerland;^20^ the Tasmanian Longitudinal Respiratory Health Study (TAHS) in Australia;^21^ and the Vlagtwedde–Vlaardingen (VlaVla) study in the Netherlands^22^ (appendix pp 2–4). We included all participants with information on lung function from spirometry, smoking status, BMI, and asthma diagnosis status from at least two visits. We excluded a single observation from an 82-year-old participant in the VlaVla study, as it was the only observation above 80 years and did not allow for a meaningful analysis of lung function trends in this age group; this participant provided data at 63 and 79 years and the final sample included participants aged 4–80 years.

All studies were approved by local ethics review boards and participants (or their parents or legal guardians) gave written informed consent. No ethical approval was sought for this pooled analysis because all patient-level datasets were anonymised, and the analyses performed were consistent with the objectives of the original studies.

### Procedures

FEV_1_, FVC, and FEV_1_/FVC were assessed by forced spirometry according to existing recommendations at the time of each study and visit, and only technically acceptable manoeuvres were included in the current analysis. The VlaVla study did not collect FVC, but slow vital capacity, and therefore contributed only to FEV_1_ models. Current asthma diagnosis and smoking status at each study visit were obtained from validated questionnaires and harmonised to maintain inferential comparability.^23^ BMI was obtained from physical examination and standardised according to WHO classification^24^, ^25^, ^26^ to define obesity status. We also combined repeated measures of current asthma diagnosis, smoking status, and obesity status to create time-fixed individual variables for each, categorised as persistent (the risk factor was present at all visits for a participant), transient (the risk factor changed status across visits for a participant), and never (the risk factor was absent at all visits for a participant; ie, persistent *vs* transient *vs* never had asthma, persistent *vs* transient *vs* never smoker, and persistent *vs* transient *vs* never had obesity). The appendix (p 14) provides full details on the procedures.

### Statistical analysis

All analyses were done separately for male and female participants, based on self-reported sex assigned at birth in each study, and for each lung function parameter expressed as absolute values (in litres, L), standardised for cohort study and height at each year of age (appendix p 15).

To depict the contribution of each study to the accelerated cohort, we used a bubble graph and histogram with absolute numbers, using observations (ie, person-visits) as the unit of analysis in 5-year age groups, allowing multiple contributions per participant. To describe the sample characteristics, we used univariate descriptive statistics (absolute and relative frequencies, and mean and SD) of sociodemographic characteristics and risk factors, counting the individual as the unit, overall and by cohort. We also plotted the frequency (expressed as percentage, with 95% CIs) of current asthma, active smoking, and obesity per year of age in the accelerated cohort, using the observations (ie, person-visits) as the unit of analysis, in which each participant could contribute to multiple ages. The trends were smoothed with use of locally estimated scatterplot smoothing with a span of 0·4.

To derive lung function trajectories over the life course, we implemented cross-classified three-level linear regression models. All models accounted for the complex data structure, whereby observations (level 1) were nested within individuals (level 2), who were simultaneously cross-classified by cohort study and birth cohort (ie, year of birth, in 10-year intervals as previously done;^8^ level 3). We included random intercepts at the individual, cohort study, and birth cohort levels to capture variation at each of these levels. We included current asthma status (yes or no), smoking status (yes, no, or former), and obesity status (yes or no), as measured at each visit (level 1), as fixed-effect covariates, and modelled them as interactions with natural cubic splines for age at each observation to allow for flexible modelling of age-dependent effects. Full model estimates are provided in the appendix (pp 20–21). We tested models ranging from one to six knots and determined the optimal number of knots using the elbow method (ie, the point in the Bayesian Information Criteria curve beyond which the decrease in the curve became less pronounced; appendix p 19).

We obtained lung function values (mean and SD) predicted by the models at each year of age from age 4 to 80 years). These estimates were then used to visually illustrate lung function trajectories (with 95% CIs) over the life course for the accelerated cohort. To compare with individual cohort studies, we created lung function trajectories separately for each study and plotted the corresponding predicted values on the same graph.

As sensitivity analyses, we derived the lung function trajectories: (1) excluding the VlaVla study to ensure FEV_1_ and FVC trajectories used the same sample; (2) weighting observations using covariate balancing propensity scores to account for participant loss to follow-up in each study, under the assumption that the losses were completely at random (ie, participants who had missing data were a random subset of the complete sample) or at random (ie, the probability that an observation was missing depended on observed participant characteristics; appendix p 16);^27^ and (3) restricting to participants who had never been diagnosed with asthma, never smoked, and never had obesity to account for potential residual confounding by these factors in the main analytical approach.

To identify the ages at which the derived trajectories exhibited breakpoints (eg, a transition from a steep increase to a moderate increase, or from an increase to a decrease) and to investigate the potential presence of plateau phases, we applied the Bayesian Estimator of Abrupt Change, Seasonal Change, and Trend model^28^ to the lung function values predicted in the previous models. We did the following: (1) obtained the ages (with 95% credible intervals [CrIs]) at which lung function exhibited breakpoints (defined by a magnitude of a change in trend >0·1 and probability of breakpoint >0·5); (2) calculated the mean (with 95% CIs) of the predicted slope within each interval demarcated by two breakpoints, to estimate the magnitude of lung function increase and decrease within each interval; (3) assessed the potential presence of plateau phases, defined by a probability greater than 50% of the slope being 0 and sustained for a minimum of 1 year; and (4) interpreted peak lung function as occurring within the 95% CrI of the breakpoint with the highest lung function values.^28^

To test the potential effects of asthma and smoking on lung function trajectories, we repeated the main analyses, including trajectories, breakpoints, and plateaus, stratified by the fixed categories of never had asthma versus persistent asthma and, in a separate stratified analysis, never smoker versus persistent smoker. All analyses were done with use of the complete dataset, as no missing values were present, in R (version 4.3.1), including the CBPS package (version 0.23)^29^ and Rbeast package.^28^

### Role of the funding source

The funders of the study had no role in study design, data collection, data analysis, data interpretation, or writing of the report.

## Results

**Figure 1:**
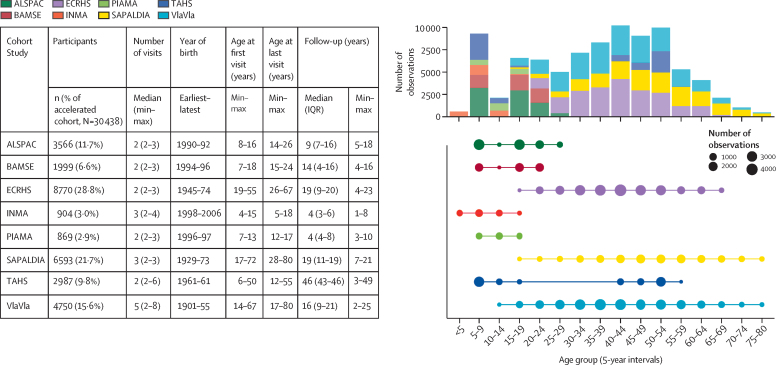
Summary of participant visits, ages, and follow-up times* according to source cohort study, and contribution of each cohort study to the accelerated cohort (absolute observation numbers) by 5-year age groups In the bubble graph and histogram, the unit of analysis is the observation (ie, person-visit). Min=minimum. Max=maximum. *This information might not match the source study characteristics, as this table reports data for participants included in the current analysis only. See the Methods section and appendix (pp 2–5) for details.

**Figure 2:**
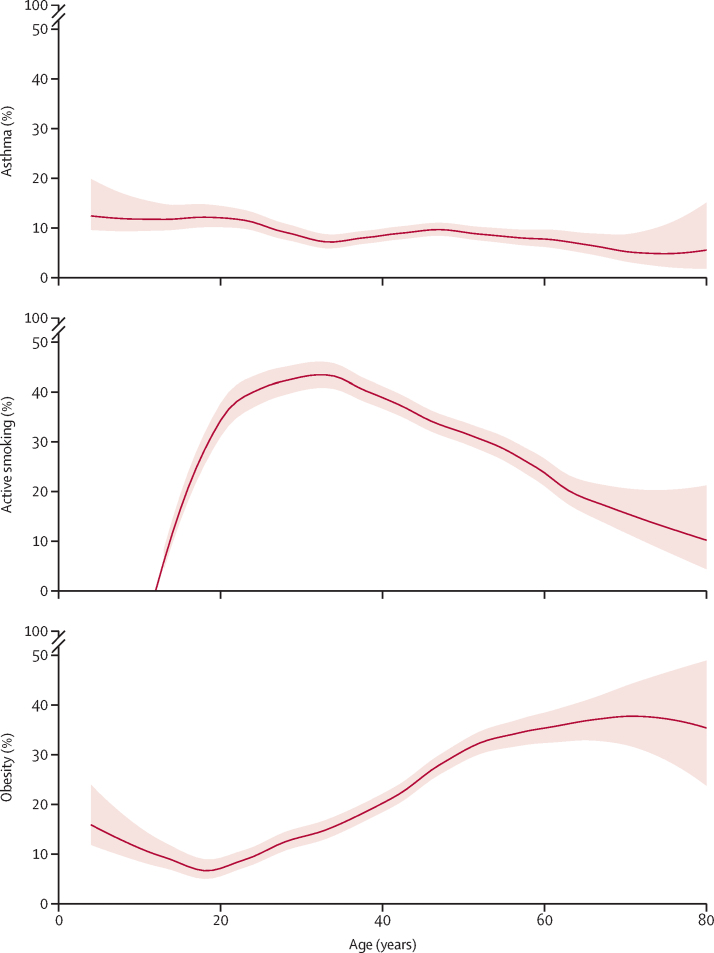
Frequency of current asthma, active smoking, and obesity by year of age in the accelerated cohort* Frequency (%) is represented by the red solid line, with 95% CIs shown in pink. Current asthma, active smoking, and obesity trends were smoothed with use of locally estimated scatterplot smoothing with a span of 0·4, applied separately to the proportions and their 95% CIs. Frequency values by year of age are provided in the appendix (pp 17–18). *The unit of analysis is the observation (ie, person-visit). Each participant contributed in at least 2 years and up to 8 years, depending on the number of visits (observations).

The accelerated cohort included 30 438 participants, born between 1901 and 2006, who provided a total of 87 666 observations (range 2–8 per participant; figure 1, appendix p 5). There were minor differences between included and excluded participants, heterogeneous by study cohort (appendix pp 6–13). Mean age of the accelerated cohort was 26 (SD 16) years (age range 4–80 years) and the cohort comprised 15 703 (51·6%) female participants and 14 735 (48·4%) male participants (table). Figure 1 shows the contribution of each study to each 5-year age interval. The frequency of current asthma ranged from 2·8% to 27·6%, depending on the year of age, and overall was highest in younger participants; the frequency of active smoking ranged from 2·9% to 52·9% and was highest in young to middle adulthood; and the frequency of obesity ranged from 2·2% to 45·5% and was highest in older adults (figure 2, appendix pp 17–18). 2056 (6·8%) of 30 438 participants had persistent asthma, 5788 (19·0%) were persistent smokers, and 2792 (9·2%) had persistent obesity (table).

**Table:** Participant characteristics*, overall and by study

		**All participants (N=30 438)**	**ALSPAC (n=3566)**	**BAMSE (n=1999)**	**ECRHS (n=8770)**	**INMA (n=904)**	**PIAMA (n=869)**	**SAPALDIA (n=6593)**	**TAHS (n=2987)**	**VlaVla (n=4750)**
**Demographics**										
Age, years		26 (16)	9 (1)	11 (4)	34 (7)	6 (2)	8 (1)	41 (12)	7 (3)	33 (11)
Sex†										
	Female	15 703 (51·6%)	2023 (56·7%)	1089 (54·5%)	4515 (51·5%)	464 (51·3%)	455 (52·4%)	3391 (51·4%)	1510 (50·6%)	2256 (47·5%)
	Male	14 735 (48·4%)	1543 (43·3%)	910 (45·5%)	4255 (48·5%)	440 (48·7%)	414 (47·6%)	3202 (48·6%)	1477 (49·4%)	2494 (52·5%)
**Asthma, smoking, and obesity status at first entry point to the present analysis**‡										
Asthma		3020 (9·9%)	355 (10·0%)	572 (28·6%)	975 (11·1%)	119 (13·2%)	40 (4·6%)	294 (4·5%)	423 (14·2%)	242 (5·1%)
Never smoker		18 681 (61·3%)	3551 (99·6%)	1930 (96·5%)	3795 (43·3%)	902 (99·8%)	869 (100%)	3061 (46·4%)	2950 (98·8%)	1623 (34·2%)
Former smoker		3945 (13·0%)	0	0	1910 (21·8%)	0	0	1515 (23·0%)	18 (0·6%)	502 (10·6%)
Active smoker		7812 (25·7%)	15 (0·4%)	69 (3·5%)	3065 (34·9%)	2 (0·2%)	0	2017 (30·6%)	19 (0·6%)	2625 (55·3%)
Obesity		3847 (12·6%)	428 (12·0%)	215 (10·8%)	1124 (12·8%)	134 (14·8%)	62 (7·1%)	839 (12·7%)	161 (5·4%)	884 (18·6%)
**Asthma, smoking, and obesity status over the life course**§										
Never had asthma		25 672 (84·3%)	2993 (83·9%)	1276 (63·8%)	7191 (82·0%)	731 (80·9%)	801 (92·2%)	6016 (91·2%)	2255 (75·5%)	4409 (92·8%)
Transient asthma		2710 (8·9%)	329 (9·2%)	151 (7·6%)	831 (9·5%)	135 (14·9%)	51 (5·9%)	368 (5·6%)	583 (19·5%)	262 (5·5%)
Persistent asthma		2056 (6·8%)	244 (6·8%)	572 (28·6%)	748 (8·5%)	38 (4·2%)	17 (2·0%)	209 (3·2%)	149 (5·0%)	79 (1·7%)
Never smoker		14 972 (49·2%)	2937 (82·4%)	1397 (69·9%)	3480 (39·7%)	893 (98·8%)	806 (92·8%)	2805 (42·5%)	1400 (46·9%)	1254 (26·4%)
Transient smoker		9678 (31·8%)	344 (9·6%)	379 (19·0%)	3565 (40·6%)	9 (1·0%)	20 (2·3%)	2619 (39·7%)	910 (30·5%)	1832 (38·6%)
Persistent smoker		5788 (19·0%)	285 (8·0%)	223 (11·2%)	1725 (19·7%)	2 (0·2%)	43 (4·9%)	1169 (17·7%)	677 (22·7%)	1664 (35·0%)
Never had obesity		20 769 (68·2%)	2834 (79·5%)	1672 (83·6%)	5774 (65·8%)	675 (74·7%)	791 (91·0%)	4389 (66·6%)	1616 (54·1%)	3018 (63·5%)
Transient obesity		6877 (22·6%)	518 (14·5%)	257 (12·9%)	2030 (23·1%)	130 (14·4%)	50 (5·8%)	1432 (21·7%)	1269 (42·5%)	1191 (25·1%)
Persistent obesity		2792 (9·2%)	214 (6·0%)	70 (3·5%)	966 (11·0%)	99 (11·0%)	28 (3·2%)	772 (11·7%)	102 (3·4%)	541 (11·4%)

Data are mean (SD) or n (%). Ethnicity data were not shared for this analysis because of some local ethical restrictions.

* The unit of analysis in this table is the individual (ie, each participant counted only once).

† Assigned at birth.

‡ The age at which participants were included for the first time in the present analysis was different for each study and participant, and ranged from 4 to 72 years of age.

§ The life course for each participant in this study corresponds to the period from the first to the last time participants were included in the analysis, the duration of which ranged from 1 to 48 years (median 16 [IQR 9–20] years).

**Figure 3:**
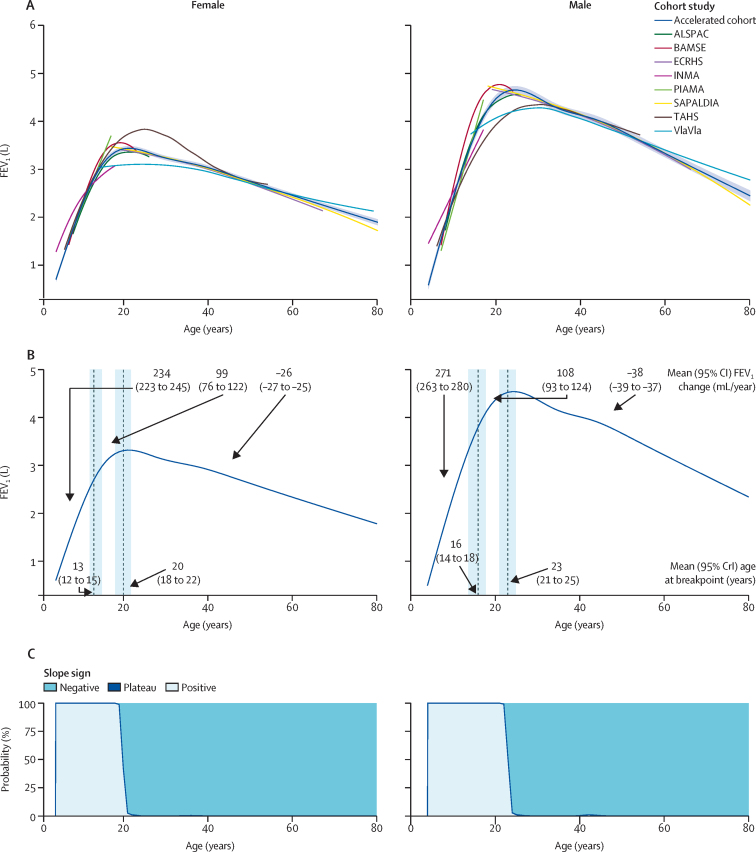
Sex-specific FEV_1_* trajectories during the life course (A) FEV_1_ trajectories in each cohort study and in the accelerated cohort, with 95% CIs for the accelerated cohort. (B) Mean age (with 95% CrIs) at lung function breakpoints and mean lung function change (with 95% CIs) between breakpoints. FEV_1_ is expressed in litres (L) and change in FEV_1_ is expressed in mL/year. Vertical dashed lines indicate the breakpoints, blue shading indicates 95% CrIs, and arrows indicate the mean FEV_1_ change between breakpoints or the mean age at breakpoints. (C) Probability of positive change (light blue), plateau (dark blue line), and negative change (blue) at each year of age of the trajectory. 95% CrI=95% credible interval. *Height-standardised and study-standardised (appendix p 15).

**Figure 4:**
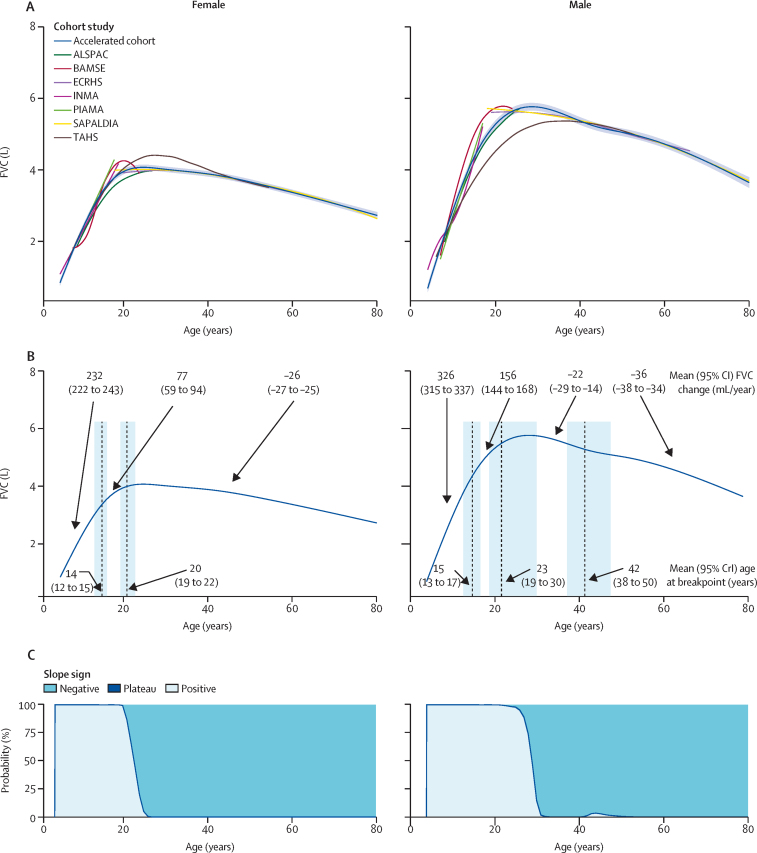
Sex-specific FVC* trajectories during the life course (A) FVC trajectories in each cohort study and in the accelerated cohort, with 95% CIs for the accelerated cohort. (B) Mean age (with 95% CrIs) at lung function breakpoints and mean lung function change (with 95% CIs) between breakpoints. FVC is expressed in litres (L) and change in FVC is expressed in mL/year. Vertical dashed lines indicate the breakpoints, blue shading indicates 95% CrIs, and arrows indicate the mean FVC change between breakpoints or the mean age at breakpoints. (C) Probability of positive change (light blue), plateau (dark blue line), and negative change (blue) at each year of age of the trajectory. 95% CrI=95% credible interval. FVC=forced vital capacity. *Height-standardised and study-standardised (appendix p 15).

In the accelerated cohort, FEV_1_ and FVC trajectories showed rapid increases in childhood followed by a phase of slower increase and a decrease in adulthood (figure 3A, figure 4A; predicted values [mean and SD] by year of age and sex are provided in the appendix [pp 22–24]). FEV_1_/FVC ratio decreased with age (appendix pp 22–25). Male and female participants had similar trajectory shapes. The trajectories in the accelerated cohort spanned a broader age range and were smoother than the study-specific trajectories. The individual sensitivity analyses excluding the VlaVla study (appendix p 26), weighting by study attrition (appendix p 27), and restricted to participants who had never had asthma, never smoked, and never had obesity (appendix p 28) showed the same patterns as the main analysis.

In female participants, FEV_1_ increased by a mean of 234 (95% CI 223 to 245) mL/year until age 13 (95% CrI 12 to 15) years, after which the increase slowed to 99 (76 to 122) mL/year until FEV_1_ reached a maximum at age 20 (18 to 22) years (figure 3B). After age 20 years, FEV_1_ decreased without any breakpoint during adulthood (−26 [−27 to −25] mL/year). In male participants, the predicted increase was 271 (263 to 280) mL/year until age 16 (14 to 18) years, after which the increase slowed to 108 (93 to 124) mL/year; peak FEV_1_ was reached at age 23 (21 to 25) years, after which FEV_1_ decreased (−38 [−39 to −37] mL/year; figure 3B).

In both male and female participants, the probability of a positive change (ie, increase) in FEV_1_ was 100% throughout childhood and adolescence (up to and including age 17 years), and the probability of a negative change (ie, decrease) was 100% during most years of adulthood (figure 3B, C). There was not a year or a period of years with a plateau for either sex.

Patterns of increase and decrease in FVC were similar to those for FEV_1_ (figure 4B). In female participants, FVC increased by a mean of 232 (95% CI 222 to 243) mL/year until age 14 (95% CrI 12 to 15) years, after which the increase slowed to 77 (59 to 94) mL/year until FVC reached a maximum at age 20 (19 to 22) years (figure 4B). After age 20 years, FVC decreased without any breakpoint during adulthood (−26 [−27 to −25] mL/year). In male participants, the mean predicted increase was 326 (315 to 337) mL/year until age 15 (13 to 17) years, after which the increase slowed to 156 (144 to 168) mL/year; peak FVC was reached at age 23 (19 to 30) years. the subsequent FVC decrease occurred in two phases, with an initial slow decrease (−22 [−29 to −14] mL/year) up to age 42 (38 to 50) years, followed by a more accelerated decrease thereafter (−36 [−38 to −34] mL/year). In both male and female participants, the probability of a positive change (ie, increase) in FVC was 100% throughout childhood and adolescence, and the probability of a negative change (ie, decrease) was 100% during most years of adulthood (figure 4B, C). There was not a year or a period of years with a plateau in FVC for either sex.

FEV_1_/FVC ratio decreased throughout life at varying rates depending on age and sex. In female participants, the decrease was minimal in childhood (mean change −0·7 × 10^−4^ [95% CI −0·8 × 10^−4^ to −0·5 × 10^−4^] per year) up to age 17 (95% CrI 14 to 19) years. This was followed by a more pronounced decrease occurring in two phases: −3·8 × 10^−4^ (−3·9 × 10^−4^ to −3·7 × 10^−4^) per year up to age 37 (35 to 39) years, and −3·3 × 10^−4^ (−3·4 × 10^−4^ to −3·2 × 10^−4^) per year thereafter (appendix p 25). In male participants, the initial decrease was steeper than in female participants (−2·7 × 10^−4^ [−2·8 × 10^−4^ to −2·6 × 10^−4^]), and remained at this magnitude until age 34 (30 to 37) years. The subsequent decrease occurred in two phases: −2·3 × 10^−4^ (−2·4 × 10^−4^ to −2·3 × 10^−4^) per year up to age 53 (49 to 57) years, and −3·9 × 10^−4^ (−4·1  × 10^−4^ to −3·8 × 10^−4^) per year thereafter (appendix p 25). In female participants, the probability of a negative change (ie, decrease) in FEV_1_/FVC ratio ranged between 75% and 100% during childhood and adolescence, and was virtually 100% for all years after age 20 years. In male participants, the probability of a negative change (ie, decrease) was virtually 100% during all years of age from 4 to 80 years (appendix p 25).

In the stratified analyses, we observed that both male and female participants with persistent asthma had an earlier peak in FEV_1_ than participants who had never had asthma (17 [95% CrI 15 to 19] years *vs* 20 [18 to 22] years in female participants; 19 [15 to 23] years *vs* 23 [20 to 26] years in male participants), as well as lower FEV_1_ throughout adulthood and markedly lower FEV_1_/FVC ratio across the life course versus those who had never had asthma (appendix pp 29, 33). Additionally, female participants with persistent asthma had an earlier peak in FVC than those who had never had asthma (18 [16 to 20] years *vs* 25 [21 to 29] years), as well as lower FVC throughout adulthood (appendix p 31). This difference in FVC peak between never and persistent asthma subgroups was not observed in male participants. Compared with never smoking, persistent smoking was associated with an accelerated decrease in FEV_1_ and FEV_1_/FVC ratio, starting in the mid-to-late-30s in both sexes (appendix pp 30, 32, 34). No strata showed a statistically significant plateau in any lung function parameter. Although the graphs suggested a potential FEV_1_ plateau around age 36 years in persistent smokers in both sexes (appendix p 30), this was not statistically significant: the probabilities of having FEV_1_ increase, plateau, or decrease at age 36 years were 44·5%, 0·3%, and 55·2% for female participants, and 44·2%, 0·0%, and 55·8% for male participants, respectively.

## Discussion

To our knowledge, this study is the first to derive general population-level lung function trajectories from childhood to late adulthood, and to use the accelerated cohort design to achieve this objective. The main findings were that, firstly, FEV_1_ and FVC increased in two phases, with a fast increase starting in early childhood (in this analysis, measured from age 4 years) until around 13–16 years, depending on sex and specific lung function parameter, followed by a slower increase until a peak. Secondly, FEV_1_ and FVC peaked earlier in female participants than in male participants, but later than previously reported in female individuals.^30^, ^31^, ^32^ Thirdly, we found no evidence of a plateau in FEV_1_ or FVC after the peak in these parameters, not even among people who had never had asthma or never smoked. Fourthly, the decrease in FEV_1_ and FVC began in the early-to-mid-20s, much earlier than previously described,^1^ and ranged from around 22 to 38 mL/year. And finally, FEV_1_/FVC ratio decreased throughout life, at a varying rate depending on age and sex. These results were robust across various sensitivity analyses, including when applying inverse probability weighting to mitigate potential selection bias from losses to follow-up.

The general population-based nature of all included cohorts, which reflects a diverse distribution of risk factors such as asthma, smoking, and obesity, does raise the question of whether the current results accurately represent lung function trajectories in a healthy population, to make them potentially useful as average values for comparison. Instead of limiting the analysis to healthy participants and stratifying by study and birth cohort, as factors known to influence lung function values and changes,^1^ we adopted a more efficient approach. We modelled study and birth cohort as random effects and adjusted for the time-varying factors of asthma, smoking, and obesity, and their interactions with age. To assess the robustness of this approach, we did a sensitivity analysis restricted to participants who had never had asthma, never smoked, and never had obesity. The results were almost unchanged compared with the main model, confirming the validity of our adjustments. Furthermore, the findings of lower lung function in people with asthma versus those who had never had asthma, and the excess lung function decline observed in persistent smokers versus never smokers are consistent with the existing literature^32^ and provide additional support for the robustness of our approach. Finally, the visual similarity between the observed lung function patterns in our study and those reported by the Global Lung Function Initiative, which includes more than 90 000 cross-sectional measurements from healthy non-smoker individuals across 33 countries, further reinforces the external validity of our findings.^6^

To date, the increase in FEV_1_ and FVC has been described either as linear (from around age 5 years until peak values are achieved),^30^, ^33^ or as exponential with a lag phase (from about age 4–5 years until age 10 years), a fast growth phase (from around 10–15 years), and a slow growth phase until peak values are achieved.^1^ Our results show that the initial lag period in children does not exist, at least from our starting point of age 4 years onwards, and that lung function increase is not linear, but rather has two phases: a fast growth phase, starting in early childhood, and a slower growth phase from around age 13–16 years until a peak, depending on sex and lung function parameter. It is conceivable that previous research, which was based on smaller studies and did not cover a long age span, did not have statistical power or could not model accurately enough the changes in lung function values.^4^ Our estimates also showed a larger absolute increase in lung function in male versus female participants, which is consistent with adult male individuals generally being taller than adult female individuals.

Previous research reported that female individuals reach peak lung function between ages 13 and 15 years,^30^, ^31^, ^32^ much earlier than our observed peaks at age 20 (95% CrI 18–22) years for FEV_1_ and 20 (19–22) years for FVC. This discrepancy could be explained by either a distinct distribution in genetic or environmental factors between our study (which includes eight different studies collectively covering ages 15–25 years) and previous studies (mostly based on single-country samples), by a potential birth cohort effect (which in our analysis was accounted for by including participants born between 1901 and 2006), or by the use of more sensitive analytical strategies in the present study. We also estimated that in male participants, peaks occurred at 23 (21–25) years for FEV_1_ and 23 (19–30) years for FVC; the finding of an earlier peak in lung function in female participants than in male participants is consistent with previous research.^30^, ^31^, ^32^

Our data do not support earlier reports of a plateau phase in lifelong lung function trajectory,^30^, ^31^, ^32^, ^33^, ^34^ as the probability of a plateau (ie, no change) in young adulthood was zero for both FEV_1_ and FVC in the main analysis, and predominantly zero across strata of asthma and smoking status. Some of these earlier studies suggested that such a plateau, particularly in female individuals, could extend up to age 40 years.^32^, ^34^ This discrepancy might arise because these previous reports did not capture the slow rate of increase that takes place from adolescence until early adulthood, due to either an absence of data or, again, less sensitive analytical strategies.^30^, ^31^, ^32^, ^33^, ^34^ Our study is larger, more heterogeneous (five of the eight individual studies contributed participant data for ages 20–40 years), and was based on measures at more ages than previous analyses. It could be argued that both our analytical approach and previous approaches tended to favour population summaries over individual trends, resulting in an inability to identify a potential plateau within specific population subgroups.^35^ However, our stratified analyses restricted to people who had never had asthma or never smoked did not show a plateau phase either.

Consistent with the absence of a plateau, we found that FEV_1_ and FVC started to decrease immediately after reaching the peak, in both male and female participants. The decrease ranged from 22 to 38 mL/year, depending on sex and the parameter. This is consistent with previous research, in which the decrease ranged from 10 to 50 mL/year, albeit with the decreases starting earlier in the present analysis than reported previously.^4^ We also observed that both FEV_1_ and FVC in female participants, and FEV_1_ in male participants, decreased linearly without any breakpoint, in contrast to previous theoretical models that proposed an initial slow decrease followed by an accelerated decrease.^1^, ^33^ This pattern was observed only for FVC in male participants in our analysis.

We found that FEV_1_/FVC ratio decreased throughout life. Notably, the pattern of decrease in FEV_1_/FVC ratio varied by sex. It is known that preschool children have higher expiration capacity relative to total lung volume than older children and adults; thus, FEV_1_/FVC ratio is physiologically higher in younger children.^36^ The finding of a less steep FEV_1_/FVC decline for female participants compared with male participants up to age 17 years is consistent with the concept of sex-related dysanapsis (ie, disproportionate scaling of airway dimensions to lung volume).^37^ Additionally, after puberty, female individuals might experience hormonal changes, such as oestrogen fluctuations, that affect airway function, as well as differences in susceptibility to respiratory diseases compared with male individuals.^37^ Together, these factors contribute to a distinct pattern of lung function decline in male and female individuals across the lifespan.

In this analysis, the observed fast growth in lung function already taking place from age 4 years, and the absence of a plateau phase in early adulthood, suggest that the risk of adult chronic respiratory disease is strongly influenced by the peak achieved. This indicates the importance of using spirometry early in life to identify individuals at risk, and reinforces the need for respiratory health promotion from early childhood through to early adulthood to support lung function growth and prevent low peak lung function.^13^ Our results also provide strong support for approaches that allow re-analysis of existing health megadata to establish more precise reference data and eventually test how and when early-life factors can affect health and disease later in life.

The main strength of our study is that the design and analysis maximised sample size and external validity, and minimised the extent of study attrition, potential survival bias, and birth cohort effect. Our study also includes some limitations. Although ethnicity data were not available for data sharing, we acknowledge, based on the previous cohort study reports,^15^, ^16^, ^17^, ^18^, ^19^, ^20^, ^21^, ^22^ that all studies, including those in Australia, included primarily participants of self-reported European White ancestry, thus limiting generalisability to other populations. This limitation extends beyond the current study, as most trajectory analyses to date have focused on European-ancestry populations. Future studies should aim to address this critical gap in knowledge, as recently emphasised by our group.^13^ Secondly, the pooling of several studies and the need to use values standardised within year of age, by height and study, limit the interpretation of absolute values of lung function, although changes in lung function over time should not have been affected. Thirdly, differences in spirometry equipment between studies might have caused systematic variations in lung function values; however, considering that multiple studies contributed results, the direction of changes over time was unlikely to be affected. Fourthly, the control of missing data due to loss to follow-up was based on a subset of the initial cohorts, as only data from participants with spirometry measurements were available to the study coordinators. However, given that the collection of spirometry data in each study was based on site-specific and age-specific protocols rather than participant characteristics, any potential bias due to such missing data is expected to be minimal. Finally, examining the role of other risk or protective factors of lung function growth and decline, such as environmental or occupational exposures, genetics, childhood infections, low birthweight, and socioeconomic or lifestyle factors, was beyond the scope of this study because of the difficulties in harmonising and sharing data from different cohorts. However, we acknowledge that these factors are likely to influence the trajectories and should be another focus of future research.

In conclusion, we found that FEV_1_ and FVC increased non-linearly during childhood, were already at a rapid growth rate by early childhood in male and female individuals, reached a peak at around age 20 years in female individuals and age 23 years in male individuals, and started to decrease thereafter without a plateau phase. The FEV_1_/FVC ratio decreased throughout life at a varying rate depending on age and sex. These results provide an essential, but previously unavailable, framework to assess and monitor lung health over the life course, which could facilitate early prevention and treatment. Considering that lung function is a marker of both respiratory and non-respiratory health, spirometry checking at all life stages has the potential to enhance overall human health across the lifespan.

### Contributors

### Data sharing

Individual participant data pooled for the current study cannot be shared with third parties due to clauses in data sharing agreements for each individual cohort study. Data can be requested for each cohort study at the following URLs: for ALSPAC, https://www.bristol.ac.uk/alspac/; for BAMSE, https://ki.se/en/imm/research/bamse-project; for ECRHS, https://www.ecrhs.org/; for INMA, https://www.proyectoinma.org/en/; for PIAMA, https://piama.iras.uu.nl/en/about-piama/; for SAPALDIA, https://www.swisstph.ch/en/projects/project-detail/project/sapaldia-program; for TAHS, https://tahs.com.au/; and for VlaVla, https://research.rug.nl/en/datasets/vlagtwedde-vlaardingen-study-vlavla. Queries can be sent to the corresponding author of this paper (judith.garcia@isglobal.org) and the Coordination and Research Management Office of the Projects Unit of ISGlobal (research.management@isglobal.org). The statistical analysis plan will be available online (https://dataverse.csuc.cat/dataverse/ISGlobal) from the date of publication of this paper.

## Declaration of interests

AA has received personal lecture fees from AstraZeneca, Chiesi, GSK, Menarini, Sanofi, Zambon, Roche, and Aerogen; his institution has received grants from GSK and AstraZeneca. SCD has received personal lecture fees from AstraZeneca, outside the submitted work; her institution has received grants from AstraZeneca and GSK. JWD has received personal lecture fees from GSK, Chiesi, Pulmonx, and AstraZeneca; his institution has received grants from GSK, AstraZeneca, Chiesi, the UK National Institute for Health and Care Research (NIHR), and Southmead Hospital Charity. GHK receives grant support to his institution from ZonMw (Vici grant), the Netherlands Lung Foundation, Health Holland, the EU, Vertex, Teva Netherlands, and GSK, outside the submitted work; his institution has received fees for consultancy or presentations from AstraZeneca, Sanofi, Boehringer Ingelheim, and PureIMS, outside the submitted work. EM has received lecture or advisory board fees from ALK, AstraZeneca, Chiesi, and Sanofi, outside the submitted work. JP's institution has received grants from AstraZeneca and GSK. JAW has received personal lecture or consulting fees from AstraZeneca, EpiEndo, GSK, Boehringer Ingelheim, Recipharm, Gilead, Novartis, Roche, Pieris, Pulmatrix, Empiricio, and Sanofi; her institution has received grants from AstraZeneca, Boehringer Ingelheim, Chiesi, GSK, Novartis, Genentech, and 37 Clinical. RF has received personal lecture fees from AstraZeneca and Chiesi, outside the submitted work; her institution has received grants from AstraZeneca, Menarini, GSK, Instituto de Salud Carlos III, the European Research Council, EU Horizon 2020, the Spanish Society of Pneumology and Thoracic Surgery, the Serra Húnter Professorship Programme, the European Respiratory Society, Chiesi, and Sanofi. JG-A, MdlH, A-EC, SA, DB, UG, TG, RG, MI, CI, AJ, SK, BL, NP-H, LS-M, MT, DV, JMV, MdV, EHW, GW, and DJ declare no competing interests. IE was unreachable at the time of final submission and therefore their declaration is unknown.
