# Supplementary material for: General population-based lung function trajectories over the life course: an accelerated cohort study
Source: Lancet Respir Med. 2025 Jul;13(7):611–22. doi: 10.1016/S2213-2600(25)00043-8 (PMC12209707; doi:10.1016/S2213-2600(25)00043-8)
Supplement: Supplementary appendix [file mmc1.pdf]

THE LANCET
Respiratory Medicine

Supplementary appendix

This appendix formed part of the original submission and has been peer reviewed. We post it as supplied by the authors.

Supplement to: Garcia-Aymerich J, de las Heras M, Carsin A-E, et al. General population-based lung function trajectories over the life course: an accelerated cohort study. *Lancet Respir Med* 2025; published online May 15. https://doi.org/10.1016/S2213-2600(25)00043-8.

GENERAL POPULATION-BASED LUNG FUNCTION TRAJECTORIES OVER THE LIFE COURSE. AN ACCELERATED COHORT STUDY

SUPPLEMENTARY METHODS

- **Text S1. Cohort studies full description**
- **Figure S1. Study flowchart**
- **Table S1. Comparison of characteristics between included and excluded participants at the first entry point to the present analysis*, overall (a) and by study (b-h)****
- **Text S2. Procedures (full version)**
- **Text S3. Standardisation of lung function within each year of age by height and study**
- **Text S4. Generation of inverse probability-of-censoring weights**

SUPPLEMENTARY RESULTS

- **Table S2. Frequency (% and 95% CI) of risk factors by year of age.**
- **Figure S2. Selection of the optimal number of knots to build the mixed models to derive lung function trajectories.**
- **Table S3. Multivariable mixed linear model estimates, by lung function parameter and sex.**
- **Table S4. Predicted yearly value (and SD) of FEV_1 (in litres), FVC (in litres) and FEV_1/FVC (ratio) in males and females from 4 to 80 years of age.**
- **Figure S3. Sex-specific FEV_1/FVC* trajectories during life course. Panel A: in each cohort study and in the accelerated cohort (with its 95% CI); Panel B: age (95% CrI) at lung function breakpoints and mean (95% CI) lung function change between breakpoints; Panel C: probability of positive change (light grey), plateau (black) and negative change (dark grey) at each year of the trajectory.**
- **Figure S4. FEV_1 S4. Sex-specific FEV_1 trajectories during life course in each cohort study and in the accelerated cohort (with its 95% CI), after excluding Vla/Vla study.**
- **Figure S5. Sex-specific FEV_1, FVC and FEV_1/FVC trajectories during life course in the accelerated cohort (with its 95% CI), after inverse probability-of-censoring weighting.**
- **Figure S6. Sex-specific FEV_1, FVC and FEV_1/FVC trajectories during life course in the accelerated cohort (with its 95% CI), after restricting to participants with never asthma, smoking or obesity.**
- **Figure S7. Sex-specific FEV_1 trajectories during life course in the accelerated cohort (with its 95% CI), stratified by asthma (a) and smoking (b).**
- **Figure S8. Sex-specific FVC trajectories during life course in the accelerated cohort (with its 95% CI), stratified by asthma (a) and smoking (b).**
- **Figure S9. Sex-specific FEV_1/FVC trajectories during life course in the accelerated cohort (with its 95% CI), stratified by asthma (a) and smoking (b).**

SUPPLEMENTARY REFERENCES

Supplementary Text S1. Cohort studies full description

ALSPAC. The UK Avon Longitudinal Study of Parents and Children birth cohort, UK[1,2,3]

Pregnant women resident in Avon, UK with expected dates of delivery between 1st April 1991 and 31st December 1992 were invited to take part in the study. 20,248 pregnancies have been identified as being eligible and the initial number of pregnancies enrolled was 14,541. Of the initial pregnancies, there was a total of 14,676 fetuses, resulting in 14,062 live births and 13,988 children who were alive at 1 year of age. When the oldest children were approximately 7 years of age, an attempt was made to bolster the initial sample with eligible cases who had failed to join the study originally. As a result, when considering variables collected from the age of seven onwards (and potentially abstracted from obstetric notes) there are data available for more than the 14,541 pregnancies mentioned above: The number of new pregnancies not in the initial sample (known as Phase I enrolment) that are currently represented in the released data and reflecting enrolment status at the age of 24 is 906, resulting in an additional 913 children being enrolled (456, 262 and 195 recruited during Phases II, III and IV respectively). The phases of enrolment are described in more detail in the cohort profile paper and its update (see footnote 5 below). The total sample size for analyses using any data collected after the age of seven is therefore 15,447 pregnancies, resulting in 15,658 fetuses. Of these 14,901 children were alive at 1 year of age. Of those, 8672 had data available at 8 years and were eligible for the present study.

Study data were collected and managed using REDCap electronic data capture tools hosted at the University of Bristol[4]. REDCap (Research Electronic Data Capture) is a secure, web-based software platform designed to support data capture for research studies.

Please note that the study website contains details of all the data that is available through a fully searchable data dictionary and variable search tool (see http://www.bristol.ac.uk/alspac/researchers/our-data/).

Ethical approval for the study was obtained from the ALSPAC Ethics and Law Committee and the Local Research Ethics Committees. Informed consent for the use of data collected via questionnaires and clinics was obtained from participants following the recommendations of the ALSPAC Ethics and Law Committee at the time.

BAMSE. The Children Allergy, Milieu, Stockholm, Epidemiology (BAMSE) study, Sweden[5]

The population-based birth cohort BAMSE [Barn/Children, Allergy, Milieu, Stockholm, Epidemiology] recruited 4,089 infants from inner-city, urban, and suburban districts of Stockholm, Sweden between February 1994 and November 1996 and followed subjects from birth until around 24 years. [6,7] Briefly, information on demographics, lifestyle characteristics, and exposures was obtained from parental questionnaires administered at recruitment, and follow-up questionnaires were answered by parents at ages 1, 2, 4, 8, 12, and 16 years. At age 24 years, participants themselves answered the questionnaire. Prebronchodilator spirometry was determined at 8, 16, and 24 years following the American Thoracic Society/European Respiratory Society (ATS/ERS) recommendations. All parents (at inclusion, 4, and 8 years) and participants (at age 16 and 24 years) signed their informed consent, under the Helsinki Declaration. BAMSE was approved by the Ethics Committee of Karolinska Institutet (Ref 2016/1380-31/2), Stockholm.

ECRHS. The European Community Respiratory Health Survey (ECRHS), Europe and Australia[8]

Between 1991-1993 (ECRHS1), participants aged 20 to 44 years were randomly selected from the population (population-based arm) and complemented with a sample of subjects with asthma-related symptoms (symptomatic arm). Two follow-up surveys took place approximately 10 years (ECRHS2) and 20 years (ECRHS3) later. All participants answered a detailed questionnaire and underwent a clinical visit at each occasion, where spirometry and blood sample collection were performed. The current study used data from both population-based and symptomatic arms, and included participants from Belgium, Denmark, France, Germany, Iceland, Italy, Norway, Spain, Sweden, Switzerland, and UK in Europe, and Australia.

INMA. Infancia y Medio Ambiente ("Environment and Childhood") Sabadell, Valencia, Menorca and Gipuzkoa birth cohorts, Spain[9]

The Infancia y Medio Ambiente (Environment and Childhood, INMA) cohort was created to study associations between environmental exposures and the physical, social, and intellectual development of Spanish children from conception onwards. INMA is a network of seven birth cohorts with participating families living in/near Ribera d'Ebre, Menorca, Granada, Valencia, Sabadell, Asturias, and Gipuzkoa, covering distinct regions of Spain in terms of environmental and cultural exposures. Between 1997 and 2008, pregnant women were recruited in the first trimester of pregnancy in regular prenatal care visits. All recruited participants completed study visits during pregnancy, at birth, and when the child reached 4 years of age. After the 4-year visit, each child was regularly followed up; however, the intervals between visits

depended on the cohort. For this analysis, only children from Gipuzkoa, Sabadell, Valencia, and Menorca were included as these are cohorts that included repeated lung function measurements using spirometry. More specifically, INMA Gipuzkoa had spirometry measures available performed by children at 4,7, and 11 years of age, INMA Sabadell performed spirometry at 4,7, and 9 years, INMA Valencia at 7 and 10 years, and INMA Menorca at 11, 14, and 18 years of the children's age. At every study visit, the child's asthma status, body mass index (BMI), passive smoking exposure and starting at the 18 year visit also active smoking exposure, and exact age were assessed.

PIAMA. The Prevention and Incidence of Asthma and Mite Allergy study, the Netherlands[10]

Pregnant women from communities in the North, West, and Central regions of the Netherlands were recruited in 1996/97 and a total of 3963 new-borns were included into the study. Data have been collected by questionnaires during pregnancy, at 3 months post-partum, annually till age 8 and thereafter at ages 11, 14, 17 and 20. Lung function measurements according to ATS/ERS criteria were performed in subsets of children at ages 8, 12 and 16. The study was approved by the respective Medical Ethics Committees and informed (parental) consent was obtained from all participants.

SAPALDIA. The Swiss study on Air Pollution and Lung Disease in adults, Switzerland[11]

SAPALDIA began in 1991 to specifically study the air pollution impact on respiratory health. It is a cohort of adults aged 18 to 60 years from population registries in eight communities in Switzerland, representing the three largest language groups (German, French, Italian) as well as different levels of air pollution and degrees of urbanisation. Participants underwent spirometry and answered a detailed questionnaire on respiratory health, allergies, smoking history, and lifestyle factors at baseline (year 1991) and four follow-up (years 2002, 2011, 2017 and 2022) examinations.

TAHS. The Tasmanian Longitudinal Health Study, Australia[12]

The Tasmanian Longitudinal Health Study began in 1968 when 8,583 children born in 1961 and attending school in Tasmania were enrolled (99% of the population). At age 7 years (baseline), the children underwent pre-bronchodilator (BD) spirometry and parents completed a detailed questionnaire. In 1974, a follow-up survey of all children from the 1968 cohort was undertaken. A total of 7380 (87.3%) were traced and completed another respiratory survey (average age 13 years). Of these 7380, a stratified random sample of 851 was selected according to symptoms of cough and wheeze in 1968 and 1974 (wheeze only, cough only, both wheeze and cough and neither symptom in both surveys) for a clinical follow-up including spirometry. In 1979 (average age 18 years) another survey was conducted of the stratified random sample from 1974. Of the 851 who were eligible, 85% (723) were traced to an address and, of these traced participants, 658 (91%) completed brief postal questionnaires. Those respondents were then invited for a clinical study that included a detailed respiratory questionnaire and clinical examination, which included the performance of spirometry. Of the 658 invited for the clinical study, only 218 (33%) completed the full study (spirometry and questionnaires); 92 (14%) completed the questionnaire only; and the remaining 348 (53%) declined the invitation. The next follow-up study occurred in the 2003-2005 period when participants were in their 5th decade of life. It involved a postal survey of all participants traced from the original 1968 cohort (average age 43 years) and a clinical study of a sub sample of participants (average age 45 years). We traced 7,562 (88.1%) of the original 1968 cohort to an address and achieved a response of 5,729 (78.4%) to a postal survey. A subgroup of these respondents enriched for cases of asthma or cough reported in childhood or adulthood was invited to participate in a more detailed laboratory study. Of 2387 invited, 1405 (58.9%) took part in a full laboratory visit including both pre-and post-BD spirometry. Between 2010 and 2012, 837 participants (average age 50 years) from the 5th decade laboratory study (59.9%) took part in a full laboratory visit including spirometry. In the sixth decade follow-up (2012-2016), when the participants were 53 years old, all those from the 1968 original cohort who were alive and had up-to-date contact details were invited to complete a survey and clinical investigations. In this follow-up, questionnaires were completed by 3609 and pre- and post-BD spirometry was performed in 2,689 participants. 60 year follow-up is now underway.

Vla/Vla. The Vlagtwedde–Vlaardingen study, the Netherlands[13]

The Vlagtwedde-Vlaardingen study is a general population-based study on the epidemiology of pulmonary diseases in a general population of exclusively white individuals of Dutch descent. The study started in 1965, and participants had medical examinations including lung function measurements according to ATS/ERS criteria every 3 years until the final survey in 1989/1990. In Vlaardingen, only participants who were included at baseline (1965 or 1969) were approached for follow-up, whereas in Vlagtwedde new subjects aged between 20 and 65 years were invited to participate at every survey. In total, 8465 subjects

had at least one survey and the number of surveys per subject ranged from one to eight (median number of surveys per subject: five).

Supplementary Figure S1. Study flowchart

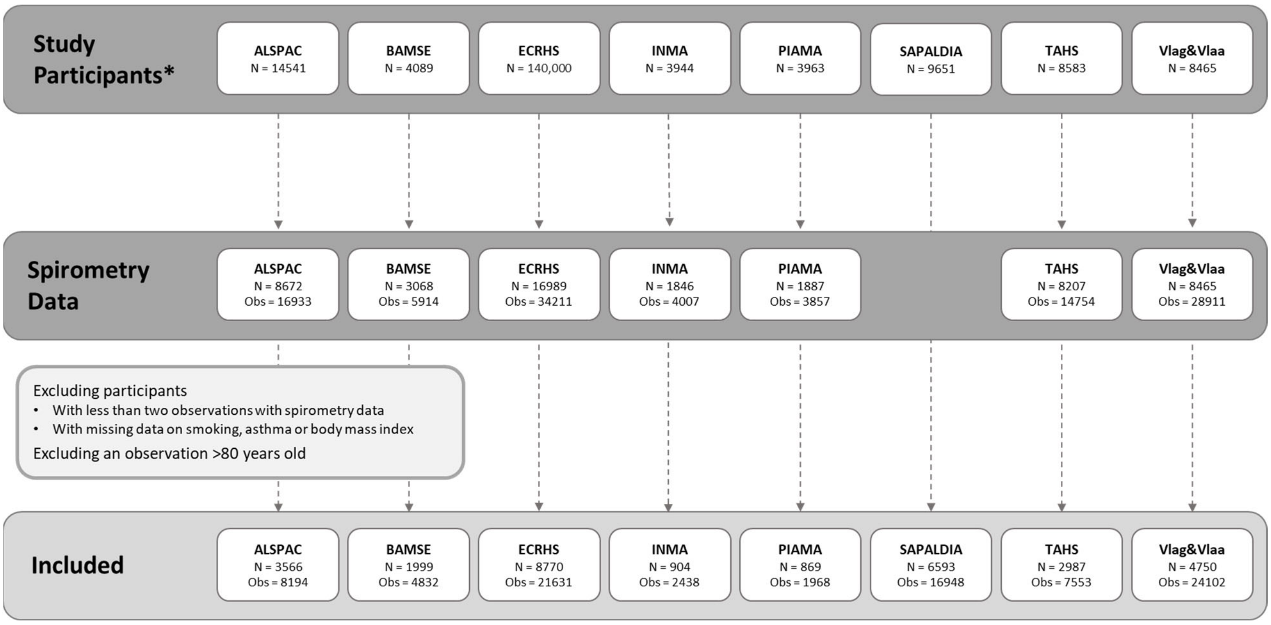

* Individual data from the total study participants was not shared with this analysis' coordinators.
** Individual data from the participants with spirometry data was shared with this analysis' coordinators (except for the SAPALDIA study), and was used for the comparison of included vs excluded participants and the inverse probability of censoring weighting.

Supplementary Table S1. Comparison of characteristics between included and excluded participants at the first entry point to the present analysis*, overall (a) and by study (b-h).**

*The unit of analysis is the individual, i.e., each subject contributed only once – the first time they were included in the present analysis, which was different for each study and participant, and ranged from 4 to 72 years of age.
** SAPALDIA not included, see Figure S1.

a. All Studies

	**Included n = 30438**	**Not Included n = 25289**	**P**
Age, mean (SD)	26 (16)	22 (16)	<0·001
Sex*, n (%)	15703 (52%)	12652 (50%)	<0·001
Never smoker, n (%)	18726 (62%)	17337 (69%)	<0·001
Former smoker, n (%)	3904 (13%)	1905 (8%)	
Active smoker, n (%)	7790 (26%)	5361 (21%)	
Asthma, n (%)	2984 (10%)	2465 (10%)	0·007
Obesity, n (%)	4139 (14%)	3056 (12%)	<0·001

*Assigned at birth

b. ALSPAC

	**Included n=3566**	**Not Included n=5106**	**p**
Age, mean (SD)	9 (1)	11 (5)	<0·001
Sex, n (%)	2023 (57%)	2475 (48%)	<0·001
Never smoker, n (%)	3561 (100%)	4805 (94%)	n.c.
Former smoker, n (%)	0 (0%)	0 (0%)	
Active smoker, n (%)	5 (0%)	150 (3%)	
Asthma, n (%)	344 (10%)	589 (12%)	<0·001
Obesity, n (%)	425 (12%)	689 (13%)	<0·001

n.c. not computable

c. BAMSE

	**Included n=1999**	**Not Included n=1069**	**p**
Age, mean (SD)	11 (4)	16 (6)	<0·001
Sex, n (%)	1089 (54%)	507 (47%)	<0·001
Never smoker, n (%)	1930 (97%)	881 (82%)	<0·001
Former smoker, n (%)	0 (0%)	55 (5%)	
Active smoker, n (%)	69 (3%)	130 (12%)	
Asthma, n (%)	572 (29%)	321 (30%)	0·436
Obesity, n (%)	215 (11%)	135 (13%)	0·135

d. ECRHS

	**Included n=8770**	**Not Included n=8219**	**p**
Age, mean (SD)	34 (7)	33 (7)	<0·001
Sex, n (%)	4515 (51%)	4375 (53%)	0·024
Never smoker, n (%)	3801 (43%)	3030 (37%)	<0·001
Former smoker, n (%)	1898 (22%)	1461 (18%)	
Active smoker, n (%)	3069 (35%)	3259 (40%)	
Asthma, n (%)	962 (11%)	815 (10%)	0·043
Obesity, n (%)	1071 (12%)	775 (9%)	0·969

e. INMA

	**Included n=904**	**Not Included n=942**	**p**
Age, mean (SD)	6 (2)	9 (2)	<0·001
Sex, n (%)	464 (51%)	458 (49%)	0·264
Never smoker, n (%)	903 (100%)	938 (100%)	n.c.
Former smoker, n (%)	0 (0%)	0 (0%)	
Active smoker, n (%)	1 (0%)	1 (0%)	
Asthma, n (%)	106 (12%)	59 (6%)	0·017
Obesity, n (%)	134 (15%)	284 (30%)	<0·001

n.c. not computable

f. PIAMA

	**Included n=869**	**Not Included n=1018**	**p**
Age, mean (SD)	8 (1)	9 (2)	<0·001
Sex, n (%)	455 (52%)	496 (49%)	0·126
Never smoker, n (%)	869 (100%)	1015 (100%)	n.c.
Former smoker, n (%)	0 (0%)	0 (0%)	
Active smoker, n (%)	0 (0%)	1 (0%)	
Asthma, n (%)	40 (5%)	34 (3%)	0·242
Obesity, n (%)	65 (7%)	81 (8%)	0·764

n.c. not computable

g. TAHS

	**Included n=2987**	**Not Included n=5220**	**p**
Age, mean (SD)	7 (3)	8 (8)	<0·001
Sex, n (%)	1510 (51%)	2482 (48%)	0·009
Never smoker, n (%)	2973 (100%)	5118 (98%)	<0·001
Former smoker, n (%)	7 (0%)	57 (1%)	
Active smoker, n (%)	7 (0%)	45 (1%)	
Asthma, n (%)	424 (14%)	599 (11%)	0·001
Obesity, n (%)	156 (5%)	340 (7%)	0·008

h. VLA/VLA

	**Included n=4750**	**Not Included n=3715**	**p**
Age, mean (SD)	33 (11)	45 (14)	<0·001
Sex, n (%)	2256 (47%)	1859 (50%)	0·021
Never smoker, n (%)	1622 (34%)	1550 (42%)	<0·001
Former smoker, n (%)	497 (10%)	332 (9%)	
Active smoker, n (%)	2619 (55%)	1775 (48%)	
Asthma, n (%)	244 (5%)	48 (1%)	<0·001
Obesity, n (%)	815 (17%)	762 (21%)	<0·001

Supplementary Text S2. Procedures (full version)

At each visit, all studies collected information on respiratory health and clinical, environmental and lifestyle factors following standardised protocols and validated questionnaires, some of which were study-specific[1,2,5,8,9,10,11,12,14]. Lung function (pre-bronchodilator FEV_1, FVC and FEV_1/FVC) was assessed by forced spirometry following existing recommendations at the time of each study and visit, and only technically acceptable manoeuvres were included. Vla/Vla study did not collect FVC and therefore contributes only to FEV_1 models. Asthma information was collected using validated questionnaires, as reported in original studies. Due to differences in definitions and methods across studies, asthma information was harmonised, keeping inferential comparability[15], to obtain a variable of current asthma at each visit (yes/no) defined as the presence of both a "self- or parental-report of doctor diagnosis" and "asthma attacks, asthma medication or wheezing within the last 12 months"[16]. Smoking status (never/former/active) was collected using validated questionnaires at each visit from 15 years of age onwards. Weight and height were obtained during physical examination. For individuals up to the age of 19 years, we computed age- and sex-specific z-scores for body mass index (z-BMI) using the World Health Organisation reference standards for the appropriate the age-group (i.e., below the age of 5 years and from 5 to 19 years)[17,18] and then defined obesity at each visit (yes/no) as z-BMI>95th percentile[19]. For individuals above 19 years, obesity was defined using a fixed cut-off of BMI≥30 kg/m^2 [20]. We also combined repeated measures of current asthma diagnosis, smoking, and obesity to create time-fixed individual variables for each, which were categorised as: persistent (the risk factor was present at all visits for a participant), transient (the risk factor changed status across visits for a participant), and never (the risk factor was absent at all visits for a participant). Only for transient and persistent smoking, we excluded observations before 14 years of age, as this was the minimum age at which smoking data were first collected.

Supplementary Text S3. Standardisation of lung function within each year of age by height and study

Lung function parameters (FEV_1, FVC and FEV_1/FVC) were standardised for height and cohort study within each year of age following these steps:

1. Generate a 'healthy' subsample after having excluded any participant reporting asthma, smoking or obesity at any period.
2. For each sex, cohort study, and lung function parameter, and using the 'healthy' subsample, run a linear regression model with lung function parameter (LF) as the outcome, and *age*, age^2 and *height* as explanatory variables. Note that we assume observations of the same individual independent.

   $$LF = \beta 0 + \beta 1 * age + \beta 2 * age^2 + \beta 3 * height$$

3. For each sex and lung function parameter, and using the 'healthy' subsample, calculate the mean LF at each year *i* (1 year bin) regardless of cohort study ($\overline{LF}_i$)
4. For each sex and lung function parameter, and in the full sample, per each observation:
   a. Estimate the predicted LF using the parameters from step 2 (β_0, β_1, β_2 and β_3)

      $$LF_{predicted} = \beta 0 + \beta 1 * age + \beta 2 * age^2 + \beta 3 * height$$

   b. Derive the residuals as

      $$LF_{residual} = LF_{observed} - LF_{predicted}$$

   c. Generate the corrected lung function by adding the residuals to the LF mean at year *i* of the healthy subsample

      $$LF_{corrected} = LF_{residual} + \overline{LF}_i$$

Supplementary Text S4. Generation of inverse probability-of-censoring weights

Inverse probability weighting (IPW) is a statistical method used to address or correct attrition bias, a form of selection bias that arises when participants who drop out of a study systematically differ from those who remain. This issue is common in cohort studies, where the participants who continue often differ in characteristics from those who drop out. To prevent biased effect estimates, IPW assigns greater weight to participants who resemble those who left the study. This approach aims to approximate the effect estimates that would have been obtained had data from the entire baseline population been available.

1. **Selection of the Population:**
We used all observations that were made available to this analysis' coordinators (second chunk of Supplementary Figure S1), regardless of the number of visits or the missingness of asthma, smoking or BMI. All observations had lung function data.

2. **Preparation of the Dataset:**
    - 2.1. We generated a new variable per participant called "participation in main analysis" with value 1 if this participant was included in the main analysis (because it had at least 2 visits and had no missing data in any of asthma, smoking and BMI) or 0 if it was not included because it had only one visit or had missing data in asthma, smoking or BMI.
    - 2.2. For each study *k* and visit *j*, to fill in the missing data for either asthma, smoking or BMI, we built 20 complete datasets using '*Multivariate Imputation by Chained Equations' (MICE)* via the 'mice' R package[21] with the following variables as part of the chained equations: *cohort study, age, sex, smoking status, asthma status, BMI, and birth year group*. Lung function variables were not included in this process.

3. **Covariate Balancing Propensity Score Calculation:**
For each study *k*, visit *j*, and complete (imputed) dataset, we built a logistic regression model to predict the probability of "participation in main analysis," using as predictors the same variables mentioned above (now without missing values, as they had been imputed). As a result, each participant had a propensity score value assigned for each visit and imputed dataset.

4. **Inverse Probability Weight Calculation:**
    - 4.1. For each participant, visit and complete dataset, we calculated weights as the inverse of the propensity scores via *'CBPS'* R Package[22].
    - 4.2. The distribution of these weights was examined, and large values (e.g., >10) were winsorised if necessary to stabilize the analysis.
    - 4.3. To obtain a single weight per participant, we calculated the mean of the weights obtained in each of the 20 imputed datasets.

5. **Use of weights in the Lung function trajectory model:**
We repeated the mixed models detailed in *Methods-Statistical Analysis main manuscript* including the generated weights with the parameter *weights* from *lmer* function.

Supplementary Table S2. Frequency (% and 95% CI) of risk factors by year of age

Age (years)	N	Asthma		Smoking		Obesity	
		n	% (95%CI)	n	% (95%CI)	n	% (95%CI)
4	582	93	16 (13·2 - 19·2)			86	14·8 (12·1 - 17·9)
5	17	1	5·9 (1 - 27)			2	11·8 (3·3 - 34·3)
6	3170	433	13·7 (12·5 - 14·9)			191	6 (5·2 - 6·9)
7	991	155	15·6 (13·5 - 18)			158	15·9 (13·8 - 18·4)
8	4694	625	13·3 (12·4 - 14·3)			582	12·4 (11·5 - 13·4)
9	420	48	11·4 (8·7 - 14·8)			85	20·2 (16·7 - 24·3)
10	378	44	11·6 (8·8 - 15·3)			79	20·9 (17·1 - 25·3)
11	230	24	10·4 (7·1 - 15·1)			50	21·7 (16·9 - 27·5)
12	795	40	5 (3·7 - 6·8)			56	7 (5·5 - 9)
13	606	63	10·4 (8·2 - 13·1)			27	4·5 (3·1 - 6·4)
14	104	8	7·7 (3·9 - 14·4)	3	2·9 (1 - 8·1)	7	6·7 (3·3 - 13·2)
15	3014	331	11 (9·9 - 12·1)	254	8·4 (7·5 - 9·5)	254	8·4 (7·5 - 9·5)
16	2256	540	23·9 (22·2 - 25·7)	306	13·6 (12·2 - 15)	116	5·1 (4·3 - 6·1)
17	622	119	19·1 (16·2 - 22·4)	172	27·7 (24·3 - 31·3)	37	5·9 (4·3 - 8·1)
18	323	9	2·8 (1·5 - 5·2)	171	52·9 (47·5 - 58·3)	20	6·2 (4 - 9·4)
19	360	19	5·3 (3·4 - 8·1)	154	42·8 (37·8 - 47·9)	21	5·8 (3·8 - 8·8)
20	411	19	4·6 (3 - 7·1)	192	46·7 (41·9 - 51·5)	9	2·2 (1·2 - 4·1)
21	984	142	14·4 (12·4 - 16·8)	373	37·9 (34·9 - 41)	68	6·9 (5·5 - 8·7)
22	1933	533	27·6 (25·6 - 29·6)	554	28·7 (26·7 - 30·7)	174	9 (7·8 - 10·4)
23	1330	160	12 (10·4 - 13·9)	410	30·8 (28·4 - 33·4)	150	11·3 (9·7 - 13·1)
24	1726	186	10·8 (9·4 - 12·3)	521	30·2 (28·1 - 32·4)	249	14·4 (12·8 - 16·2)
25	1129	122	10·8 (9·1 - 12·8)	398	35·3 (32·5 - 38·1)	142	12·6 (10·8 - 14·6)
26	884	67	7·6 (6 - 9·5)	398	45 (41·8 - 48·3)	102	11·5 (9·6 - 13·8)
27	955	70	7·3 (5·8 - 9·2)	446	46·7 (43·6 - 49·9)	106	11·1 (9·3 - 13·2)
28	930	72	7·7 (6·2 - 9·6)	439	47·2 (44 - 50·4)	91	9·8 (8 - 11·9)
29	1114	79	7·1 (5·7 - 8·8)	516	46·3 (43·4 - 49·3)	136	12·2 (10·4 - 14·3)
30	1368	102	7·5 (6·2 - 9)	579	42·3 (39·7 - 45)	180	13·2 (11·5 - 15·1)
31	1348	121	9 (7·6 - 10·6)	589	43·7 (41·1 - 46·4)	198	14·7 (12·9 - 16·7)
32	1420	116	8·2 (6·9 - 9·7)	590	41·5 (39 - 44·1)	213	15 (13·2 - 17)
33	1556	110	7·1 (5·9 - 8·5)	664	42·7 (40·2 - 45·1)	234	15 (13·3 - 16·9)
34	1460	115	7·9 (6·6 - 9·4)	627	42·9 (40·4 - 45·5)	225	15·4 (13·6 - 17·4)
35	1490	112	7·5 (6·3 - 9)	619	41·5 (39·1 - 44·1)	234	15·7 (13·9 - 17·6)
36	1652	130	7·9 (6·7 - 9·3)	703	42·6 (40·2 - 45)	282	17·1 (15·3 - 19)
37	1648	150	9·1 (7·8 - 10·6)	684	41·5 (39·1 - 43·9)	297	18 (16·2 - 20)
38	1706	109	6·4 (5·3 - 7·7)	680	39·9 (37·6 - 42·2)	312	18·3 (16·5 - 20·2)
39	1805	139	7·7 (6·6 - 9)	732	40·6 (38·3 - 42·8)	341	18·9 (17·2 - 20·8)
40	1798	155	8·6 (7·4 - 10)	726	40·4 (38·1 - 42·7)	362	20·1 (18·3 - 22)
41	1921	157	8·2 (7 - 9·5)	692	36 (33·9 - 38·2)	411	21·4 (19·6 - 23·3)
42	2014	163	8·1 (7 - 9·4)	770	38·2 (36·1 - 40·4)	438	21·7 (20 - 23·6)
43	2034	197	9·7 (8·5 - 11)	725	35·6 (33·6 - 37·8)	462	22·7 (20·9 - 24·6)
44	2443	238	9·7 (8·6 - 11)	834	34·1 (32·3 - 36)	605	24·8 (23·1 - 26·5)
45	2055	222	10·8 (9·5 - 12·2)	729	35·5 (33·4 - 37·6)	551	26·8 (24·9 - 28·8)
46	1748	200	11·4 (41548)	614	35·1 (32·9 - 37·4)	459	26·3 (24·2 - 28·4)
47	1654	116	7 (5·9 - 8·3)	576	34·8 (32·6 - 37·2)	418	25·3 (23·2 - 27·4)
48	1700	173	10·2 (8·8 - 11·7)	571	33·6 (31·4 - 35·9)	459	27 (24·9 - 29·2)
49	1895	196	10·3 (9·1 - 11·8)	590	31·1 (29·1 - 33·3)	591	31·2 (29·1 - 33·3)
50	1762	171	9·7 (8·4 - 11·2)	572	32·5 (30·3 - 34·7)	529	30 (27·9 - 32·2)
51	2063	174	8·4 (7·3 - 9·7)	602	29·2 (27·3 - 31·2)	667	32·3 (30·3 - 34·4)
52	2479	217	8·8 (7·7 - 9·9)	692	27·9 (26·2 - 29·7)	952	38·4 (36·5 - 40·3)
53	2138	220	10·3 (9·1 - 11·6)	648	30·3 (28·4 - 32·3)	747	34·9 (32·9 - 37)
54	1520	134	8·8 (7·5 - 10·3)	439	28·9 (26·7 - 31·2)	492	32·4 (30·1 - 34·8)
55	1126	68	6 (4·8 - 7·6)	363	32·2 (29·6 - 35)	362	32·1 (29·5 - 34·9)
56	1138	75	6·6 (5·3 - 8·2)	311	27·3 (24·8 - 30)	394	34·6 (31·9 - 37·4)
57	1119	88	7·9 (6·4 - 9·6)	324	29 (26·4 - 31·7)	375	33·5 (30·8 - 36·3)
58	951	54	5·7 (4·4 - 7·3)	261	27·4 (24·7 - 30·4)	322	33·9 (30·9 - 36·9)
59	976	93	9·5 (7·8 - 11·5)	251	25·7 (23·1 - 28·6)	316	32·4 (29·5 - 35·4)
60	1005	75	7·5 (6 - 9·3)	219	21·8 (19·3 - 24·4)	338	33·6 (30·8 - 36·6)
61	807	67	8·3 (6·6 - 10·4)	158	19·6 (17 - 22·5)	301	37·3 (34 - 40·7)
62	784	65	8·3 (6·6 - 10·4)	156	19·9 (17·3 - 22·8)	283	36·1 (32·8 - 39·5)

63	804	73	9·1 (7·3 - 11·3)	165	20·5 (17·9 - 23·5)	295	36·7 (33·4 - 40·1)
64	695	60	8·6 (6·8 - 11)	137	19·7 (16·9 - 22·8)	254	36·5 (33 - 40·2)
65	628	37	5·9 (4·3 - 8)	121	19·3 (16·4 - 22·5)	232	36·9 (33·3 - 40·8)
66	474	28	5·9 (4·1 - 8·4)	89	18·8 (15·5 - 22·5)	182	38·4 (34·1 - 42·9)
67	423	23	5·4 (3·7 - 8)	80	18·9 (15·5 - 22·9)	170	40·2 (35·6 - 44·9)
68	288	15	5·2 (3·2 - 8·4)	49	17 (13·1 - 21·8)	106	36·8 (31·4 - 42·5)
69	314	18	5·7 (3·7 - 8·9)	41	13·1 (9·8 - 17·2)	115	36·6 (31·5 - 42·1)
70	308	16	5·2 (3·2 - 8·3)	34	11 (42217)	118	38·3 (33·1 - 43·9)
71	273	12	4·4 (2·5 - 7·5)	37	13·6 (10 - 18·1)	102	37·4 (31·8 - 43·2)
72	207	8	3·9 (2 - 7·4)	35	16·9 (12·4 - 22·6)	77	37·2 (30·9 - 44)
73	129	2	1·6 (0·4 - 5·5)	27	20·9 (14·8 - 28·7)	42	32·6 (25·1 - 41)
74	105	5	4·8 (2·1 - 10·7)	19	18·1 (11·9 - 26·5)	36	34·3 (25·9 - 43·8)
75	103	7	6·8 (3·3 - 13·4)	15	14·6 (9 - 22·6)	35	34 (25·6 - 43·6)
76	101	4	4 (1·6 - 9·7)	8	7·9 (4·1 - 14·9)	46	45·5 (36·2 - 55·2)
77	80	5	6·2 (2·7 - 13·8)	8	10 (5·2 - 18·5)	36	45 (34·6 - 55·9)
78	80	3	3·8 (1·3 - 10·5)	6	7·5 (3·5 - 15·4)	31	38·8 (28·8 - 49·7)
79	68	2	2·9 (0·8 - 10·1)	5	7·4 (3·2 - 16·1)	20	29·4 (19·9 - 41·1)
80	45	4	8·9 (3·5 - 20·7)	7	15·6 (7·7 - 28·8)	15	33·3 (21·4 - 47·9)

Supplementary Figure S2. Selection of the optimal number of knots* to build the mixed models to derive lung function trajectories.

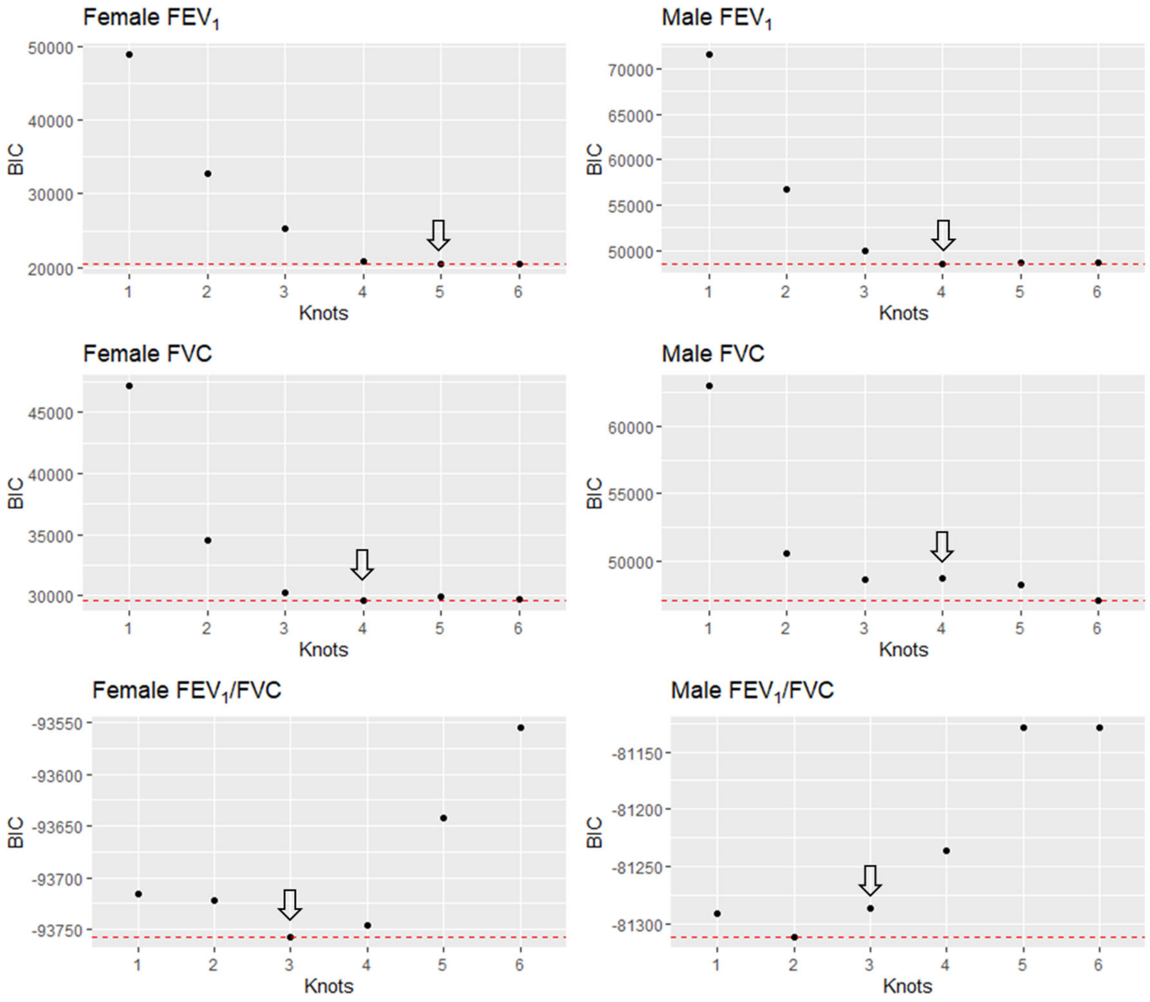

*The arrows point to selected number of knots.

Supplementary Table S3. Multivariable mixed linear models, by lung function parameter and sex.

		FEV_1				FVC				FEV_1/FVC			
		In females		In males		In females		In males		In females		In males	
Effect	Term	Coefficient (SE)	P-value*	Coefficient (SE)	P-value*	Coefficient (SE)	P-value*	Coefficient (SD)	P-value*	Coefficient (SD)	P-value*	Coefficient (SD)	P-value*
Fixed	(Intercept)	0·70 (0·02)	<0·001	0·59 (0·04)	<0·001	0·85 (0·04)	<0·001	0·70 (0·05)	<0·001	0·90 (0·00)	<0·001	0·89 (0·01)	<0·001
Fixed	Former smoker	0·03 (0·66)	0·964	3·28 (0·36)	<0·001	2·06 (1·05)	0·049	4·99 (1·26)	<0·001	-0·03 (0·06)	0·658	0·09 (0·06)	0·121
Fixed	Current smoker	0·55 (0·13)	<0·001	0·91 (0·10)	<0·001	0·26 (0·17)	0·123	-1·62 (0·24)	<0·001	0·04 (0·01)	0·008	0·05 (0·02)	0·001
Fixed	Age spline 1	2·52 (0·01)	<0·001	3·63 (0·02)	<0·001	3·15 (0·01)	<0·001	5·35 (0·03)	<0·001	-0·08 (0·00)	<0·001	-0·09 (0·00)	<0·001
Fixed	Age spline 2	2·41 (0·01)	<0·001	3·51 (0·02)	<0·001	3·05 (0·02)	<0·001	4·50 (0·02)	<0·001	-0·12 (0·00)	<0·001	-0·11 (0·00)	<0·001
Fixed	Age spline 3	2·16 (0·01)	<0·001	1·02 (0·02)	<0·001	1·36 (0·02)	<0·001	2·53 (0·03)	<0·001	-0·19 (0·00)	<0·001	-0·21 (0·00)	<0·001
Fixed	Age spline 4	0·57 (0·01)	<0·001	6·38 (0·03)	<0·001	5·07 (0·03)	<0·001	7·48 (0·05)	<0·001	-0·23 (0·00)	<0·001	-0·23 (0·01)	<0·001
Fixed	Age spline 5	4·09 (0·02)	<0·001	-0·49 (0·04)	<0·001	0·21 (0·03)	<0·001	0·69 (0·06)	<0·001				
Fixed	Age spline 6	-0·29 (0·02)	<0·001										
Fixed	Obesity	-0·12 (0·02)	<0·001	-0·08 (0·03)	0·002	-0·12 (0·03)	<0·001	-0·15 (0·04)	<0·001	-0·01 (0·00)	0·003	0·00 (0·00)	0·61
Fixed	Asthma	0·03 (0·02)	0·169	0·00 (0·02)	0·877	-0·01 (0·02)	0·813	-0·11 (0·03)	<0·001	0·00 (0·00)	0·821	-0·01 (0·00)	0·048
Fixed	Former smoker * Age spline 1	0·04 (0·65)	0·956	-3·16 (0·35)	<0·001	-1·95 (1·04)	0·06	-5·24 (1·24)	<0·001	0·03 (0·06)	0·622	-0·08 (0·06)	0·162
Fixed	Current smoker * Age spline 1	-0·55 (0·12)	<0·001	-0·96 (0·09)	<0·001	-0·27 (0·16)	0·078	1·08 (0·22)	<0·001	-0·03 (0·01)	0·021	-0·04 (0·01)	0·005
Fixed	Former smoker * Age spline 2	0·00 (0·67)	0·999	-3·35 (0·37)	<0·001	-2·04 (1·05)	0·053	-4·91 (1·27)	<0·001	0·01 (0·04)	0·742	-0·06 (0·04)	0·121
Fixed	Current smoker * Age spline 2	-0·55 (0·14)	<0·001	-1·05 (0·10)	<0·001	-0·21 (0·17)	0·239	1·75 (0·25)	<0·001	-0·05 (0·01)	<0·001	-0·06 (0·01)	<0·001
Fixed	Former smoker * Age spline 3	-0·01 (0·66)	0·984	-1·87 (0·21)	<0·001	-1·22 (0·65)	0·058	-3·37 (0·76)	<0·001	0·03 (0·12)	0·825	-0·23 (0·12)	0·056
Fixed	Current smoker * Age spline 3	-0·63 (0·13)	<0·001	-0·75 (0·06)	<0·001	-0·35 (0·09)	<0·001	0·29 (0·13)	0·029	-0·13 (0·03)	<0·001	-0·16 (0·03)	<0·001
Fixed	Former smoker * Age spline 4	-0·01 (0·39)	0·985	-6·65 (0·72)	<0·001	-4·08 (1·99)	0·041	-9·06 (2·43)	<0·001	0·01 (0·03)	0·775	-0·03 (0·03)	0·219
Fixed	Current smoker * Age spline 4	-0·43 (0·08)	<0·001	-2·06 (0·21)	<0·001	-0·46 (0·35)	0·187	3·76 (0·49)	<0·001	-0·04 (0·01)	<0·001	-0·07 (0·01)	<0·001
Fixed	Former smoker * Age spline 5	-0·27 (1·28)	0·831	-1·45 (0·17)	<0·001	-1·04 (0·51)	0·042	-2·74 (0·61)	<0·001				
Fixed	Current smoker * Age spline 5	-1·29 (0·27)	<0·001	-0·60 (0·07)	<0·001	-0·11 (0·10)	0·247	0·38 (0·14)	0·006				
Fixed	Former smoker * Age spline 6	-0·06 (0·32)	0·861										
Fixed	Current smoker * Age spline 6	-0·48 (0·08)	<0·001										

Fixed	Age spline 1 * Obesity	0·09 (0·03)	<0·001	0·02 (0·03)	0·452	0·18 (0·03)	<0·001	0·03 (0·05)	0·571	0·00 (0·00)	0·338	0·01 (0·00)	0·172
Fixed	Age spline 2 * Obesity	0·06 (0·03)	0·057	-0·03 (0·03)	0·344	-0·02 (0·03)	0·602	-0·04 (0·05)	0·375	0·04 (0·00)	<0·001	0·03 (0·00)	<0·001
Fixed	Age spline 3 * Obesity	0·04 (0·02)	0·122	0·05 (0·03)	0·084	-0·07 (0·03)	0·006	-0·12 (0·04)	0·002	0·00 (0·01)	0·903	-0·04 (0·01)	<0·001
Fixed	Age spline 4 * Obesity	0·06 (0·02)	0·001	-0·16 (0·07)	0·025	0·15 (0·07)	0·038	0·03 (0·11)	0·801	0·03 (0·01)	<0·001	0·02 (0·01)	0·007
Fixed	Age spline 5 * Obesity	0·16 (0·06)	0·006	-0·05 (0·04)	0·281	-0·14 (0·04)	<0·001	-0·12 (0·06)	0·051				
Fixed	Age spline 6 * Obesity	0·00 (0·03)	0·987										
Fixed	Age spline 1 * Asthma	-0·20 (0·02)	<0·001	-0·32 (0·04)	<0·001	-0·13 (0·03)	<0·001	-0·05 (0·05)	0·3	-0·03 (0·00)	<0·001	-0·03 (0·00)	<0·001
Fixed	Age spline 2 * Asthma	-0·15 (0·03)	<0·001	-0·28 (0·03)	<0·001	-0·06 (0·03)	0·088	-0·06 (0·05)	0·24	-0·02 (0·00)	<0·001	-0·04 (0·01)	<0·001
Fixed	Age spline 3 * Asthma	-0·24 (0·02)	<0·001	-0·32 (0·04)	<0·001	-0·26 (0·03)	<0·001	-0·19 (0·06)	0·002	-0·05 (0·01)	<0·001	-0·06 (0·01)	<0·001
Fixed	Age spline 4 * Asthma	-0·20 (0·03)	<0·001	-0·33 (0·08)	<0·001	0·02 (0·07)	0·794	0·13 (0·10)	0·208	-0·02 (0·01)	0·07	-0·01 (0·01)	0·265
Fixed	Age spline 5 * Asthma	-0·20 (0·05)	<0·001	-0·26 (0·10)	0·011	-0·13 (0·07)	0·051	-0·39 (0·13)	0·003				
Fixed	Age spline 6 * Asthma	-0·22 (0·06)	<0·001										
**Effect**	**Term**	**Coefficient**	**P-value†**	**Coefficient**	**P-value†**	**Coefficient**	**P-value†**	**Coefficient**	**P-value†**	**Coefficient**	**P-value†**	**Coefficient**	**P-value†**
Random	Subject nested to study	0·30	<0·001	0·42	<0·001	0·34	<0·001	0·45	<0·001	0·05	<0·001	0·05	<0·001
Random	Subject nested to birth cohort	$5e^{-10}$	1	$2e^{-8}$	1	$1e^{-4}$	1	$1e^{-9}$	1	$4e^{-8}$	1	$3e^{-9}$	1
Random	Birth cohort	0·02	<0·001	0·05	<0·001	0·02	<0·001	0·04	<0·001	0·01	<0·001	0·01	<0·001
Random	Study	0·06	<0·001	0·10	<0·001	0·09	<0·001	0·13	<0·001	0·01	<0·001	0·01	<0·001

Every column is a single mixed model.
*Satterthwaite's method for t test.
†ANOVA test comparing the model with and without inclusion of each random effect (one at a time).

Supplementary Table S4. Predicted yearly value (and SD) of FEV_1 (in liters), FVC (in liters) and FEV_1/FVC (ratio) in males and females from 4 to 80 years of age.

Age	Female						Male					
	FEV_1 (l)		FVC (l)		FEV_1/FVC (ratio)		FEV_1 (l)		FVC (l)		FEV_1/FVC (ratio)	
	n	Value (SD)	n	Value (SD)	n	Value (SD)	n	Value (SD)	n	Value (SD)	n	Value (SD)
**4**	284	0·6964 (0·0234)	280	0·8495 (0·0371)	280	0·8954 (0·0049)	298	0·588 (0·0407)	294	0·6964 (0·0529)	294	0·8908 (0·0068)
**5**	10	0·9624 (0·0232)	10	1·1401 (0·0369)	10	0·8955 (0·0049)	7	0·908 (0·0405)	7	1·0754 (0·0525)	7	0·8892 (0·0068)
**6**	1475	1·2261 (0·023)	1581	1·4282 (0·0367)	1468	0·8956 (0·0048)	1481	1·2262 (0·0403)	1578	1·4521 (0·0522)	1477	0·8875 (0·0068)
**7**	523	1·4849 (0·0229)	523	1·7117 (0·0366)	523	0·8957 (0·0048)	467	1·5407 (0·0402)	467	1·8242 (0·052)	466	0·8859 (0·0068)
**8**	2481	1·7365 (0·0228)	2522	1·9882 (0·0365)	2477	0·8956 (0·0048)	2147	1·8498 (0·0401)	2157	2·1894 (0·0519)	2136	0·8842 (0·0068)
**9**	224	1·9784 (0·0228)	222	2·2552 (0·0365)	219	0·8955 (0·0048)	189	2·1515 (0·0401)	185	2·5455 (0·0519)	181	0·8824 (0·0068)
**10**	187	2·2083 (0·0229)	185	2·5106 (0·0365)	185	0·8952 (0·0048)	191	2·4442 (0·0401)	187	2·8901 (0·052)	187	0·8806 (0·0068)
**11**	113	2·4238 (0·0229)	113	2·7518 (0·0366)	113	0·8948 (0·0048)	117	2·7258 (0·0401)	117	3·2209 (0·0521)	117	0·8788 (0·0068)
**12**	398	2·6223 (0·023)	400	2·9766 (0·0367)	398	0·8941 (0·0048)	394	2·9947 (0·0402)	394	3·5357 (0·0522)	393	0·8768 (0·0068)
**13**	290	2·8016 (0·0231)	298	3·1827 (0·0368)	290	0·8932 (0·0048)	302	3·249 (0·0402)	308	3·8321 (0·0524)	302	0·8747 (0·0068)
**14**	65	2·9592 (0·0231)	58	3·3676 (0·0368)	57	0·8921 (0·0049)	38	3·4869 (0·0403)	30	4·1079 (0·0525)	30	0·8725 (0·0068)
**15**	1595	3·0927 (0·0232)	1577	3·529 (0·0368)	1534	0·8907 (0·0049)	1354	3·7065 (0·0404)	1293	4·3608 (0·0525)	1271	0·8702 (0·0068)
**16**	1262	3·2005 (0·0231)	1205	3·6653 (0·0368)	1202	0·889 (0·0049)	990	3·9061 (0·0404)	929	4·589 (0·0525)	928	0·8678 (0·0068)
**17**	323	3·2841 (0·0231)	264	3·7779 (0·0368)	263	0·887 (0·0049)	297	4·0837 (0·0405)	228	4·7931 (0·0525)	227	0·8652 (0·0068)
**18**	146	3·3458 (0·0231)	28	3·8688 (0·0368)	28	0·8846 (0·0049)	177	4·2377 (0·0405)	32	4·9742 (0·0524)	32	0·8624 (0·0068)
**19**	179	3·3879 (0·023)	91	3·9399 (0·0367)	91	0·882 (0·0049)	180	4·366 (0·0405)	75	5·1334 (0·0524)	74	0·8596 (0·0068)
**20**	203	3·4128 (0·023)	94	3·9935 (0·0367)	91	0·8791 (0·0049)	204	4·4677 (0·0404)	91	5·2718 (0·0523)	90	0·8566 (0·0068)
**21**	485	3·4227 (0·023)	343	4·0315 (0·0367)	341	0·876 (0·0049)	492	4·5443 (0·0404)	304	5·3905 (0·0523)	299	0·8535 (0·0068)
**22**	1042	3·42 (0·023)	920	4·0559 (0·0367)	913	0·8727 (0·0049)	882	4·598 (0·0404)	723	5·4907 (0·0524)	721	0·8504 (0·0068)
**23**	757	3·407 (0·0231)	617	4·0688 (0·0368)	611	0·8692 (0·0049)	566	4·6312 (0·0404)	377	5·5734 (0·0524)	376	0·8471 (0·0068)
**24**	966	3·386 (0·0231)	795	4·0723 (0·0369)	787	0·8656 (0·0048)	741	4·646 (0·0404)	510	5·6397 (0·0526)	501	0·8439 (0·0068)
**25**	604	3·3592 (0·0232)	452	4·0685 (0·0369)	446	0·8618 (0·0048)	513	4·6449 (0·0404)	323	5·6908 (0·0527)	317	0·8406 (0·0068)
**26**	415	3·3291 (0·0232)	237	4·0593 (0·037)	234	0·8579 (0·0048)	459	4·6299 (0·0405)	216	5·7277 (0·0529)	208	0·8372 (0·0068)
**27**	431	3·2979 (0·0233)	221	4·0467 (0·0371)	217	0·854 (0·0048)	515	4·6035 (0·0405)	231	5·7516 (0·0531)	225	0·8339 (0·0068)

**28**	459	3·268 (0·0233)	256	4·033 (0·0372)	252	0·85 (0·0049)	460	4·5678 (0·0406)	225	5·7636 (0·0533)	218	0·8306 (0·0068)
**29**	530	3·2411 (0·0233)	317	4·0197 (0·0372)	313	0·846 (0·0049)	576	4·5251 (0·0407)	292	5·7647 (0·0534)	288	0·8272 (0·0068)
**30**	682	3·2172 (0·0232)	420	4·007 (0·0372)	416	0·8419 (0·0049)	672	4·4778 (0·0408)	355	5·7562 (0·0535)	345	0·8239 (0·0068)
**31**	684	3·1956 (0·0232)	416	3·9947 (0·0372)	413	0·838 (0·0049)	652	4·428 (0·0409)	379	5·739 (0·0535)	370	0·8207 (0·0068)
**32**	672	3·176 (0·0232)	403	3·9828 (0·0372)	399	0·834 (0·0049)	738	4·3781 (0·041)	410	5·7143 (0·0535)	405	0·8175 (0·0068)
**33**	771	3·1576 (0·0232)	454	3·971 (0·0371)	449	0·8302 (0·0049)	770	4·3303 (0·041)	406	5·683 (0·0534)	395	0·8144 (0·0068)
**34**	730	3·1398 (0·0232)	441	3·9591 (0·0371)	433	0·8264 (0·0049)	714	4·2868 (0·041)	390	5·6463 (0·0533)	381	0·8114 (0·0068)
**35**	725	3·1222 (0·0233)	431	3·9471 (0·0371)	428	0·8228 (0·0049)	756	4·2494 (0·0409)	404	5·605 (0·0532)	398	0·8085 (0·0069)
**36**	816	3·1041 (0·0233)	478	3·9346 (0·0371)	474	0·8193 (0·0049)	823	4·2175 (0·0408)	434	5·5604 (0·0531)	425	0·8057 (0·0069)
**37**	817	3·085 (0·0233)	493	3·9216 (0·0371)	484	0·816 (0·0049)	811	4·1899 (0·0407)	446	5·5133 (0·0531)	434	0·8031 (0·0069)
**38**	810	3·0643 (0·0233)	497	3·9079 (0·0371)	490	0·8129 (0·0049)	877	4·1653 (0·0407)	490	5·4649 (0·0531)	478	0·8005 (0·0069)
**39**	901	3·0416 (0·0233)	530	3·8932 (0·0371)	529	0·8099 (0·0049)	890	4·1427 (0·0407)	487	5·4162 (0·0531)	474	0·7981 (0·0068)
**40**	900	3·0169 (0·0232)	578	3·8776 (0·0372)	569	0·807 (0·0049)	873	4·1208 (0·0407)	499	5·3681 (0·0531)	483	0·7958 (0·0068)
**41**	922	2·9907 (0·0231)	619	3·8606 (0·0372)	610	0·8042 (0·0049)	986	4·0984 (0·0407)	605	5·3218 (0·0531)	599	0·7936 (0·0068)
**42**	1017	2·9632 (0·0231)	655	3·8423 (0·0372)	647	0·8015 (0·0049)	976	4·0744 (0·0407)	586	5·2783 (0·0531)	572	0·7915 (0·0068)
**43**	983	2·9346 (0·0231)	672	3·8225 (0·0371)	665	0·7988 (0·0049)	1033	4·0475 (0·0407)	703	5·2386 (0·0531)	691	0·7894 (0·0068)
**44**	1167	2·9052 (0·0231)	911	3·8012 (0·0371)	894	0·7962 (0·0049)	1239	4·0169 (0·0407)	908	5·2035 (0·053)	888	0·7872 (0·0068)
**45**	1023	2·8754 (0·0232)	717	3·7786 (0·0371)	706	0·7936 (0·0049)	1009	3·9828 (0·0407)	674	5·1724 (0·053)	662	0·7851 (0·0068)
**46**	880	2·8455 (0·0232)	575	3·7548 (0·037)	563	0·791 (0·0049)	844	3·9456 (0·0406)	554	5·1445 (0·0529)	540	0·783 (0·0068)
**47**	795	2·8156 (0·0232)	528	3·7299 (0·037)	520	0·7883 (0·0049)	841	3·9058 (0·0406)	492	5·1191 (0·0528)	481	0·7808 (0·0068)
**48**	844	2·7858 (0·0232)	551	3·7042 (0·037)	544	0·7856 (0·0049)	835	3·8638 (0·0405)	513	5·0953 (0·0528)	499	0·7785 (0·0068)
**49**	944	2·7561 (0·0231)	658	3·6776 (0·037)	655	0·7828 (0·0049)	941	3·8202 (0·0405)	624	5·0723 (0·0528)	617	0·7761 (0·0068)
**50**	844	2·7266 (0·0231)	593	3·6503 (0·037)	581	0·7799 (0·0049)	895	3·7753 (0·0406)	566	5·0494 (0·0529)	554	0·7736 (0·0068)
**51**	1019	2·6972 (0·0231)	785	3·6226 (0·0371)	749	0·7769 (0·0049)	987	3·7297 (0·0406)	697	5·0257 (0·053)	675	0·771 (0·0068)
**52**	1218	2·6679 (0·0231)	985	3·5944 (0·0371)	957	0·7738 (0·0049)	1203	3·6838 (0·0407)	943	5·0005 (0·0531)	913	0·7682 (0·0068)
**53**	1075	2·6387 (0·0231)	846	3·5659 (0·0371)	825	0·7707 (0·0049)	1027	3·6379 (0·0408)	768	4·973 (0·0532)	750	0·7652 (0·0068)
**54**	746	2·6096 (0·0232)	521	3·5371 (0·0372)	506	0·7674 (0·0049)	749	3·5922 (0·0409)	494	4·9433 (0·0533)	484	0·7622 (0·0068)
**55**	555	2·5806 (0·0232)	345	3·5081 (0·0372)	336	0·7641 (0·0049)	551	3·5467 (0·041)	327	4·9113 (0·0534)	318	0·759 (0·0068)

**56**	565	2·5518 (0·0233)	375	3·4788 (0·0373)	365	0·7607 (0·0049)	552	3·5014 (0·041)	327	4·8773 (0·0535)	316	0·7557 (0·0068)
**57**	521	2·5231 (0·0233)	336	3·4493 (0·0373)	326	0·7572 (0·0049)	577	3·4562 (0·0411)	353	4·8412 (0·0536)	341	0·7523 (0·0068)
**58**	470	2·4944 (0·0234)	326	3·4195 (0·0373)	316	0·7536 (0·0049)	459	3·4111 (0·0411)	291	4·8031 (0·0536)	277	0·7488 (0·0068)
**59**	480	2·4659 (0·0234)	313	3·3895 (0·0374)	305	0·75 (0·0049)	475	3·3661 (0·0412)	285	4·7632 (0·0537)	272	0·7452 (0·0069)
**60**	512	2·4375 (0·0234)	362	3·3593 (0·0374)	350	0·7463 (0·0049)	470	3·3213 (0·0412)	296	4·7215 (0·0538)	284	0·7414 (0·0069)
**61**	394	2·4091 (0·0235)	266	3·3289 (0·0374)	256	0·7425 (0·0049)	393	3·2766 (0·0413)	277	4·6782 (0·0539)	266	0·7376 (0·0069)
**62**	407	2·3809 (0·0235)	292	3·2983 (0·0375)	280	0·7387 (0·0049)	354	3·232 (0·0414)	234	4·6332 (0·0540)	223	0·7337 (0·0069)
**63**	357	2·3527 (0·0236)	257	3·2676 (0·0376)	248	0·7348 (0·005)	434	3·1876 (0·0416)	280	4·5867 (0·0542)	276	0·7297 (0·0069)
**64**	342	2·3246 (0·0236)	256	3·2366 (0·0377)	254	0·7308 (0·005)	342	3·1432 (0·0417)	245	4·5387 (0·0545)	235	0·7256 (0·007)
**65**	297	2·2965 (0·0237)	207	3·2055 (0·0378)	201	0·7268 (0·005)	315	3·0989 (0·042)	225	4·4894 (0·0549)	214	0·7214 (0·007)
**66**	218	2·2686 (0·0239)	157	3·1743 (0·038)	149	0·7228 (0·005)	235	3·0548 (0·0423)	154	4·4389 (0·0553)	141	0·7172 (0·007)
**67**	204	2·2407 (0·024)	147	3·1429 (0·0382)	139	0·7187 (0·0051)	198	3·0107 (0·0427)	126	4·3871 (0·0558)	115	0·7129 (0·0071)
**68**	141	2·2128 (0·0243)	109	3·1113 (0·0385)	100	0·7146 (0·0051)	129	2·9667 (0·0432)	99	4·3343 (0·0565)	90	0·7085 (0·0071)
**69**	155	2·185 (0·0245)	128	3·0797 (0·0388)	119	0·7104 (0·0052)	141	2·9227 (0·0438)	105	4·2804 (0·0573)	96	0·7041 (0·0072)
**70**	141	2·1573 (0·0249)	121	3·0479 (0·0392)	112	0·7062 (0·0053)	150	2·8788 (0·0444)	123	4·2256 (0·0582)	115	0·6996 (0·0073)
**71**	140	2·1296 (0·0253)	125	3·0161 (0·0397)	116	0·702 (0·0053)	115	2·835 (0·0452)	95	4·1700 (0·0593)	86	0·6951 (0·0074)
**72**	101	2·1019 (0·0258)	89	2·9841 (0·0401)	82	0·6977 (0·0054)	95	2·7913 (0·0461)	71	4·1136 (0·0605)	67	0·6905 (0·0075)
**73**	52	2·0743 (0·0263)	40	2·9521 (0·0407)	34	0·6935 (0·0055)	68	2·7476 (0·0471)	38	4·0565 (0·0619)	35	0·6859 (0·0076)
**74**	49	2·0467 (0·0269)	38	2·92 (0·0413)	32	0·6892 (0·0056)	48	2·7039 (0·0482)	32	3·9989 (0·0634)	30	0·6813 (0·0077)
**75**	37	2·0192 (0·0276)	32	2·8878 (0·042)	24	0·6848 (0·0057)	48	2·6603 (0·0494)	43	3·9407 (0·065)	33	0·6766 (0·0078)
**76**	47	1·9916 (0·0283)	42	2·8556 (0·0427)	38	0·6805 (0·0058)	45	2·6167 (0·0507)	37	3·8821 (0·0668)	32	0·6719 (0·008)
**77**	34	1·9641 (0·0291)	34	2·8233 (0·0435)	26	0·6761 (0·0059)	31	2·5731 (0·0521)	31	3·8231 (0·0687)	24	0·6672 (0·0081)
**78**	40	1·9366 (0·03)	34	2·791 (0·0444)	28	0·6718 (0·006)	29	2·5295 (0·0536)	28	3·7639 (0·0707)	23	0·6625 (0·0083)
**79**	34	1·9091 (0·0309)	30	2·7587 (0·0453)	27	0·6674 (0·0062)	26	2·486 (0·0552)	25	3·7046 (0·0727)	20	0·6578 (0·0084)
**80**	15	1·8816 (0·0319)	20	2·7264 (0·0462)	14	0·663 (0·0063)	22	2·4424 (0·0568)	21	3·6451 (0·0749)	19	0·653 (0·0086)

Supplementary Figure S3. Sex-specific FEV1/FVC* trajectories during life course. Panel A: in each cohort study and in the accelerated cohort (with its 95% CI); Panel B: age (95% CrI) at lung function breakpoints and mean (95% CI) lung function change between breakpoints; Panel C: probability of positive change (light grey), plateau (black) and negative change (dark grey) at each year of the trajectory.

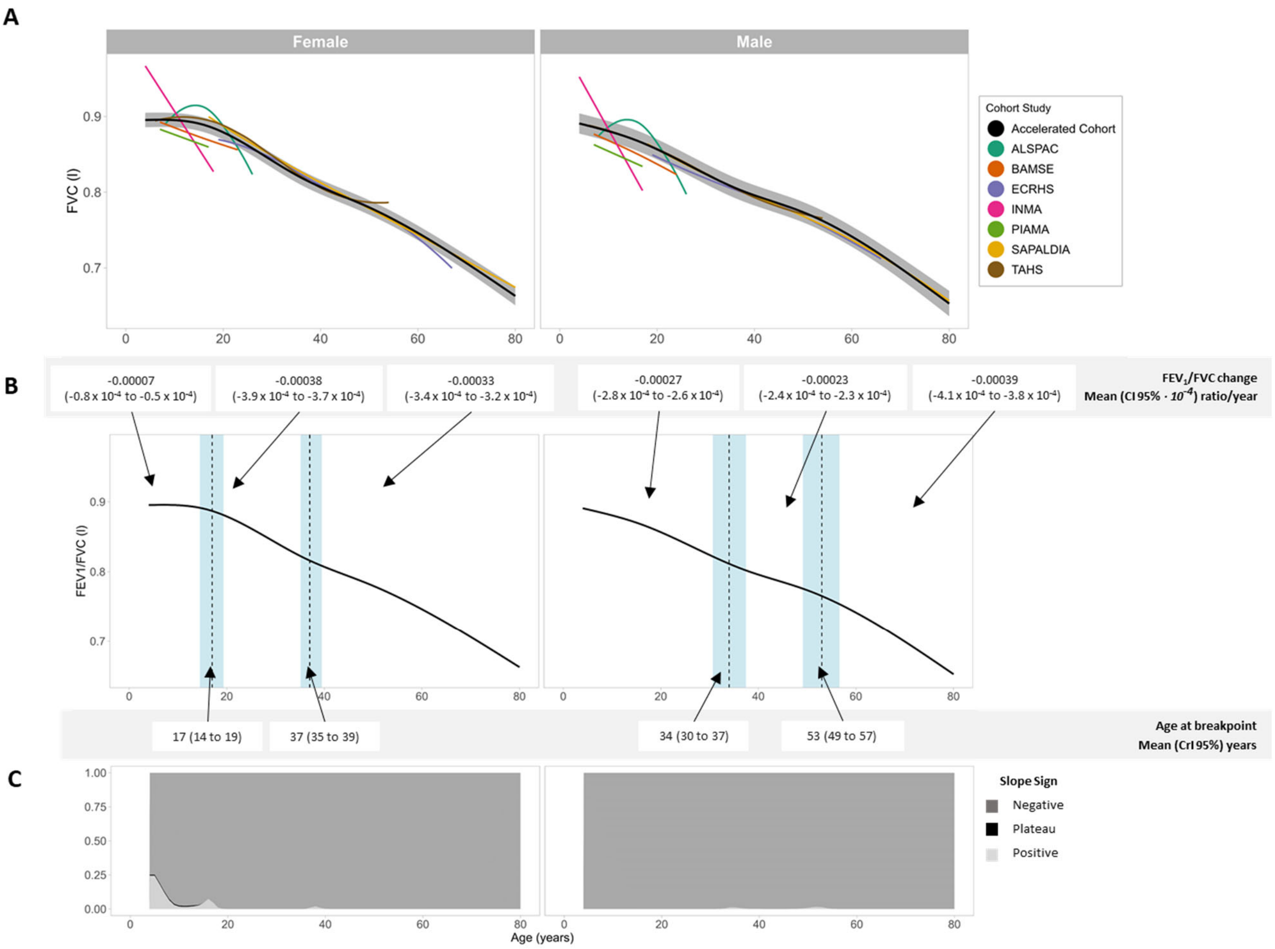

*Height- and study-standardised, see Supplementary Text S3.

Supplementary Figure S4. Sex-specific FEV_1 trajectories during life course in each cohort study and in the accelerated cohort (with its 95% CI), after excluding Vla/Vla study.

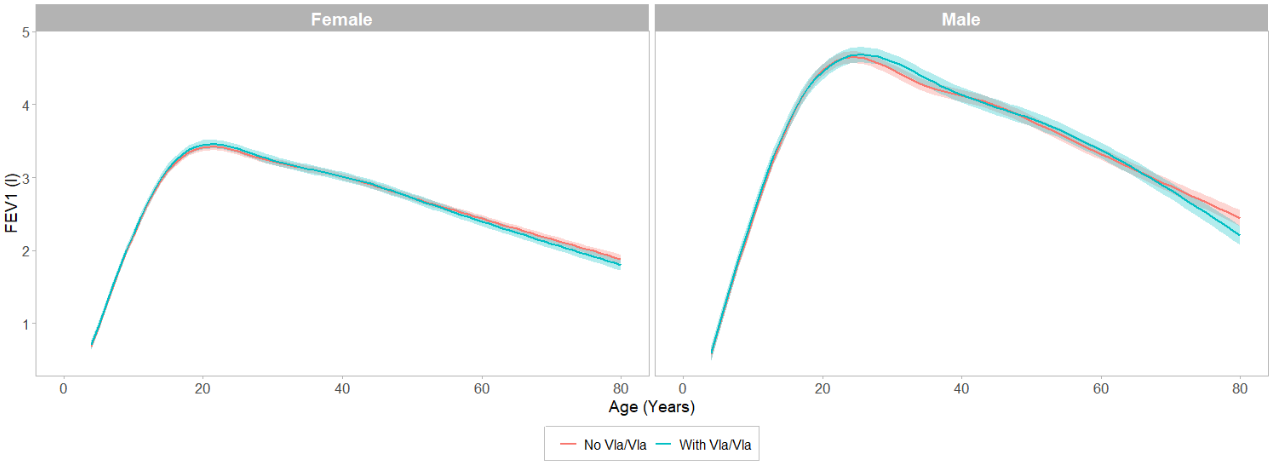

Supplementary Figure S5. Sex-specific FEV_1, FVC and FEV_1/FVC trajectories during life course in the accelerated cohort (with its 95% CI), after inverse probability-of-censoring weighting.

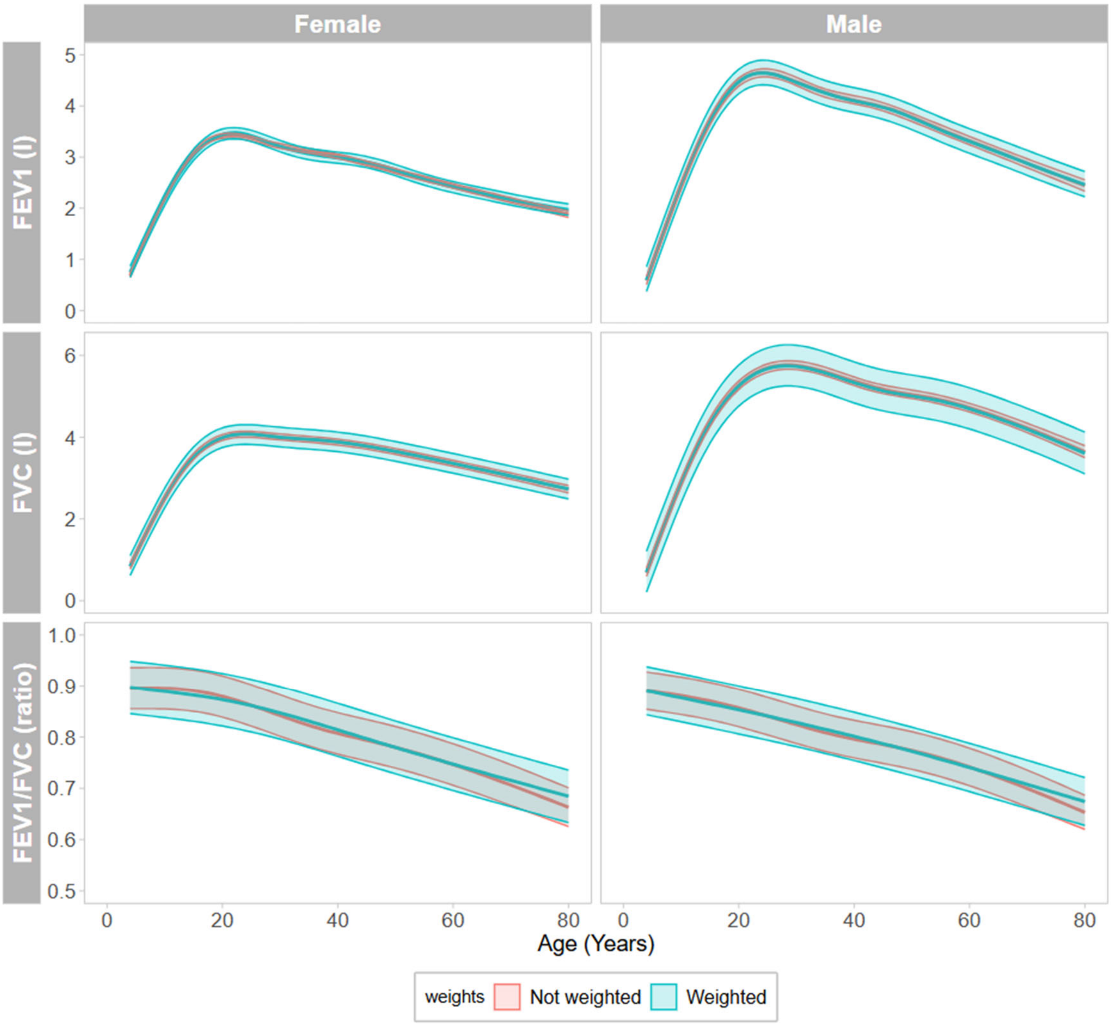

Supplementary Figure S6. Sex-specific FEV$_1$, FVC and FEV$_1$/FVC trajectories during life course in the accelerated cohort (with its 95% CI), <u>after restricting to participants with never asthma, smoking or obesity.</u>

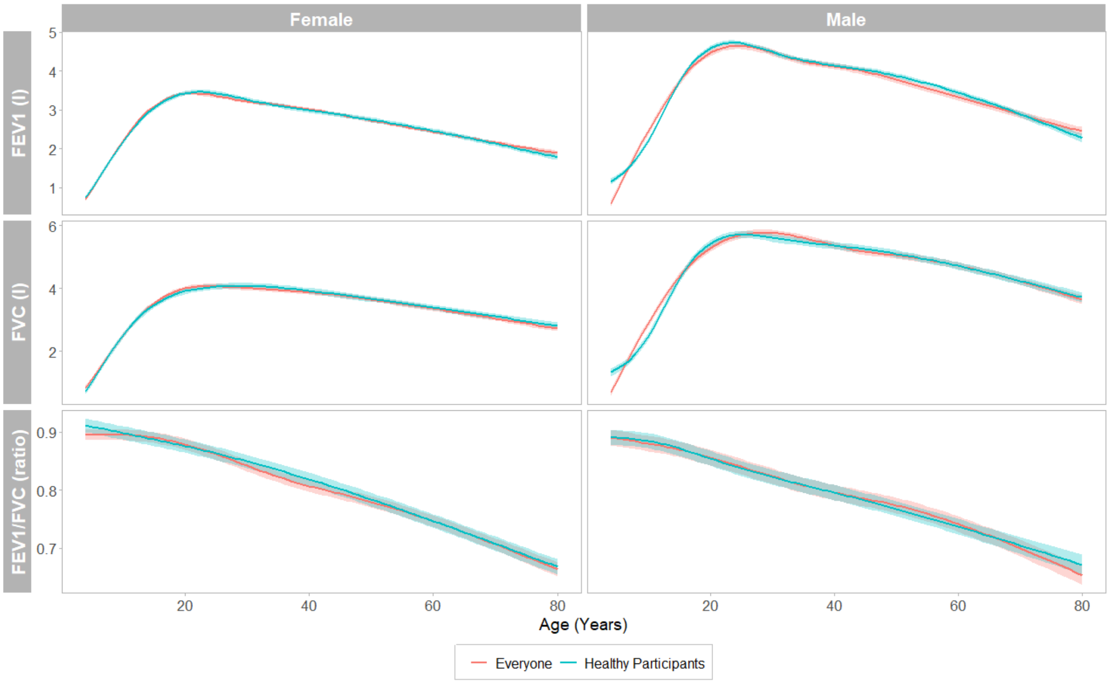

Supplementary Figure S7. Sex-specific FEV_1 trajectories during life course in the accelerated cohort (with its 95% CI), stratified by asthma (a)* and smoking (b)†.

(a)

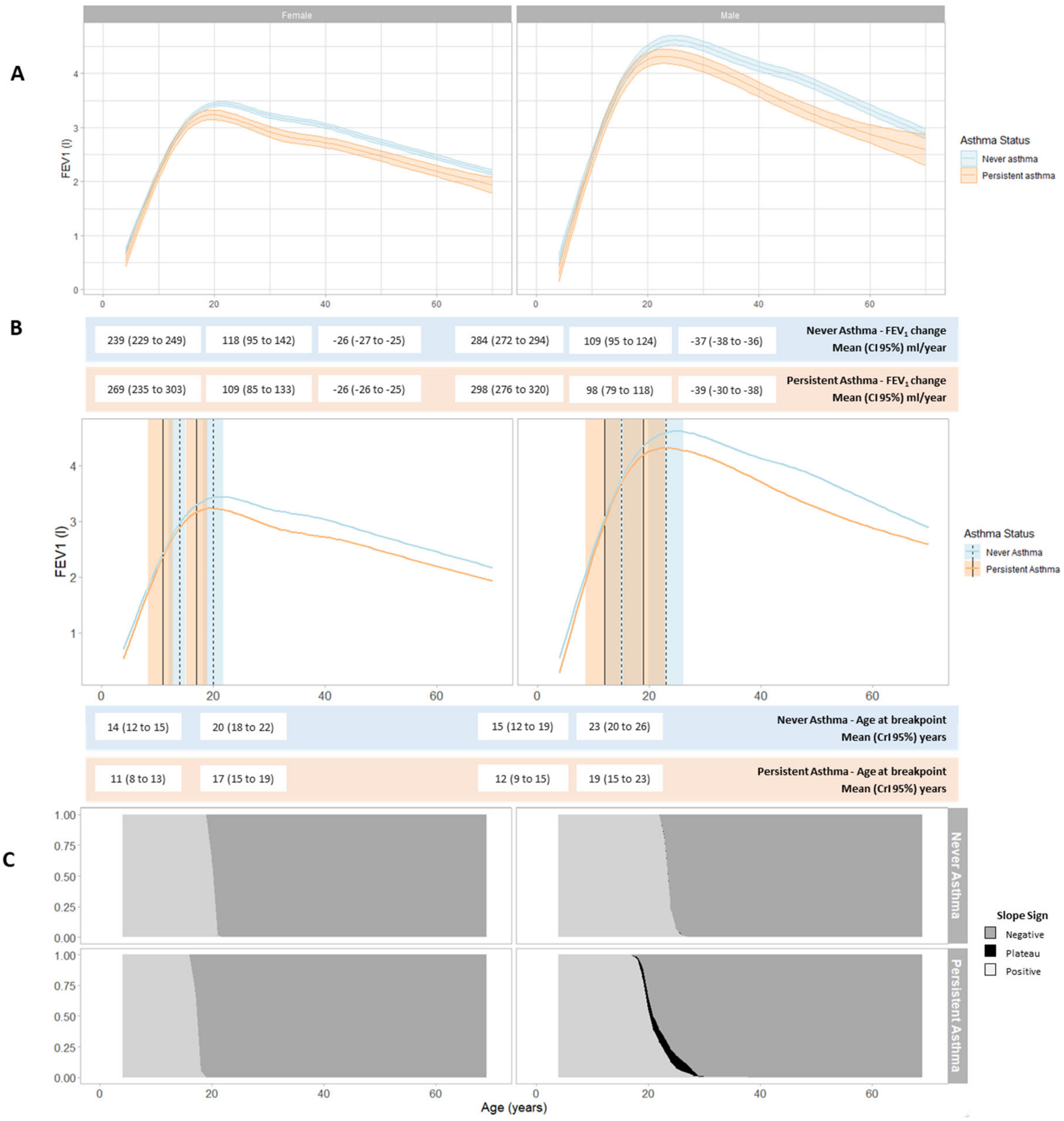

* Female: Never asthma – 13,124 (83·6%, 36,974 observations), Persistent asthma – 1,027 (6·5%, 2,550 observations); Male: Never asthma – 12,548 (85·2%, 36,739 observations), Persistent asthma – 1,029 (7·0%, 2,542 observations)

(b)

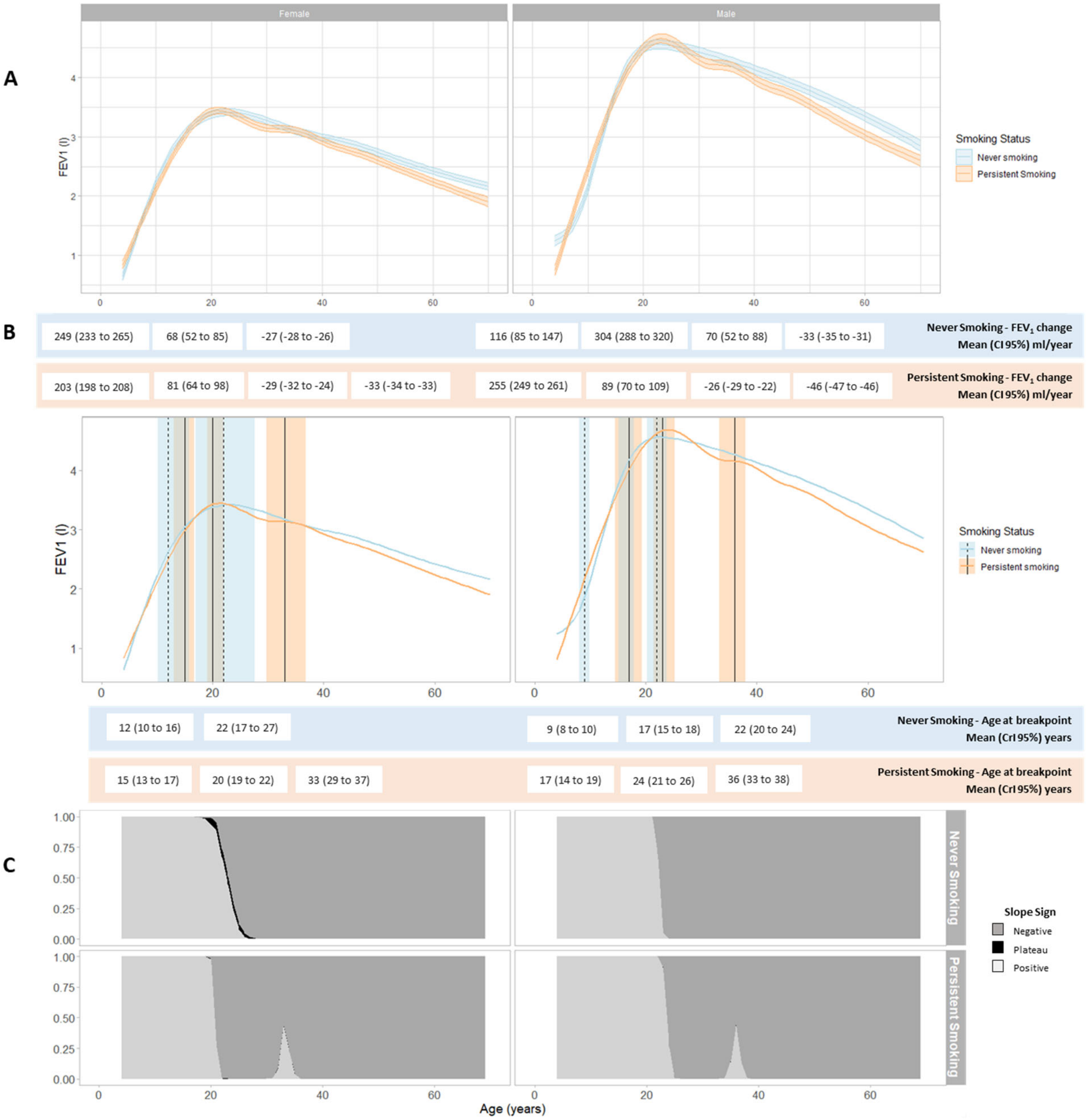

† Female: Never smoking – 6,408 (43·5%, 16,162 observations), Persistent smoking – 2,588 (16·5%, 7,077 observations); Male: Never smoking – 8,564 (54·5%, 23,304 observations), Persistent smoking – 3,200 (21·7%, 10,093 observations)

Supplementary **Figure S8.** Sex-specific FVC trajectories during life course in the accelerated cohort (with its 95% CI), stratified by asthma (a)* and smoking (b)†.

(a)

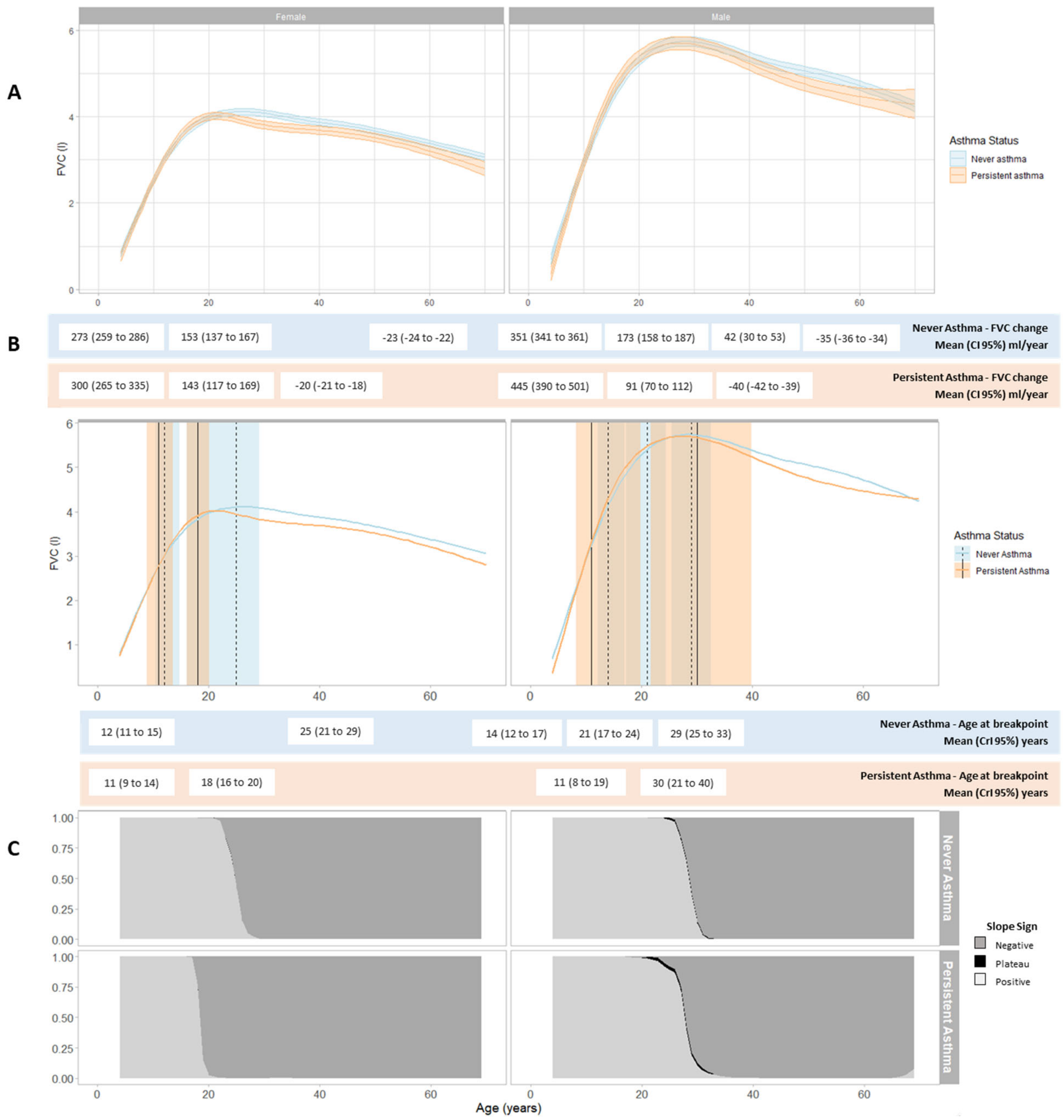

* Female: Never asthma – 11,025 (82·0%, 26,928 observations), Persistent asthma – 992 (7·4%, 2,386 observations); Male: Never asthma – 10,230 (83·6%, 24,779 observations), Persistent asthma – 984 (8·0%, 2,345 observations)

(b)

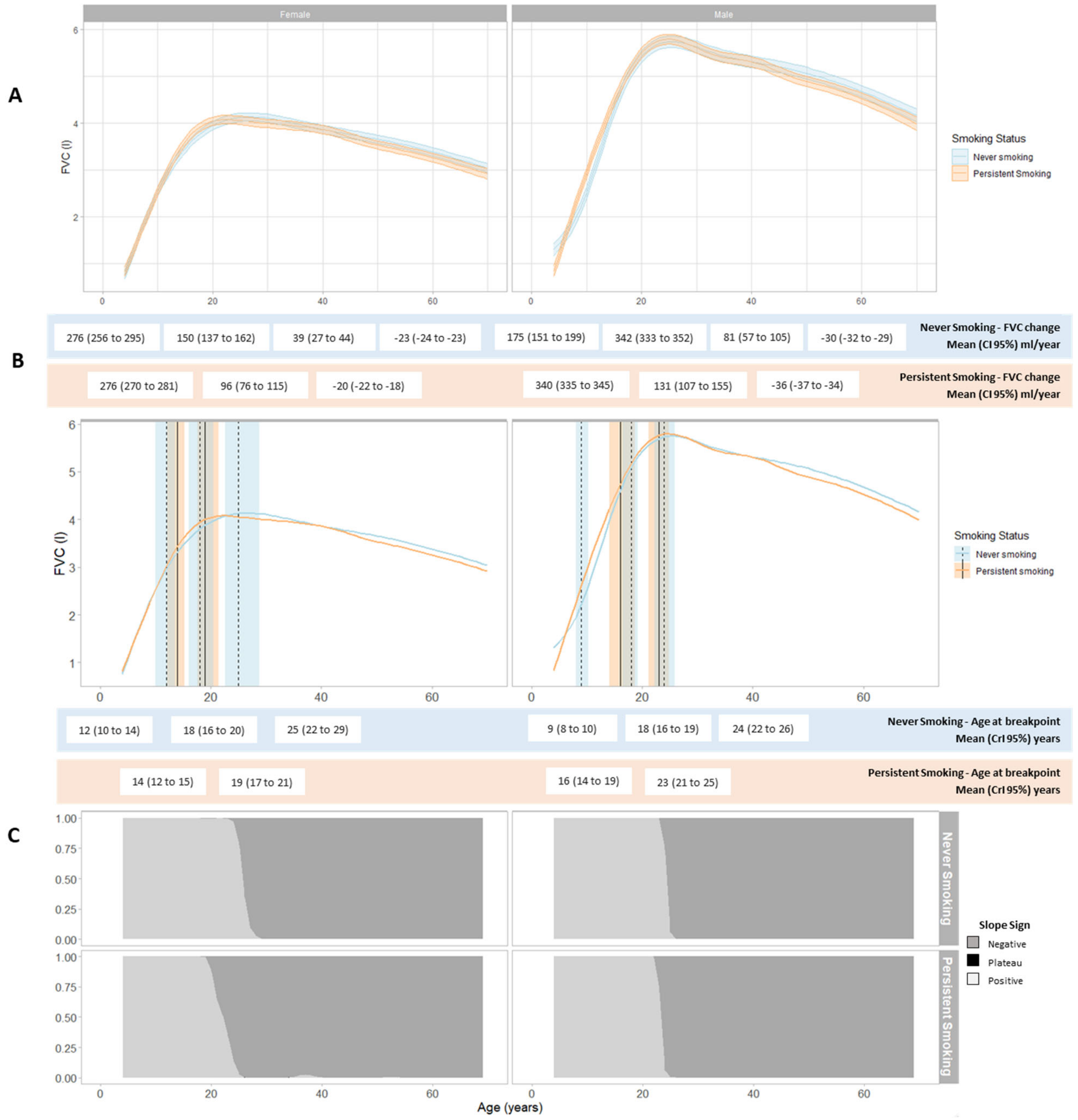

† Female: Never smoking – 7,590 (56·5%, 18,541 observations), Persistent smoking – 2,039 (15·2%, 4,729 observations); Male: Never smoking – 6,124 (50·1%, 14,799 observations), Persistent smoking – 2,082 (17·0%, 4,801 observations)

Supplementary Figure S9. Sex-specific FEV_1/FVC trajectories during life course in the accelerated cohort (with its 95% CI), stratified by asthma (a) and smoking (b).

(a)

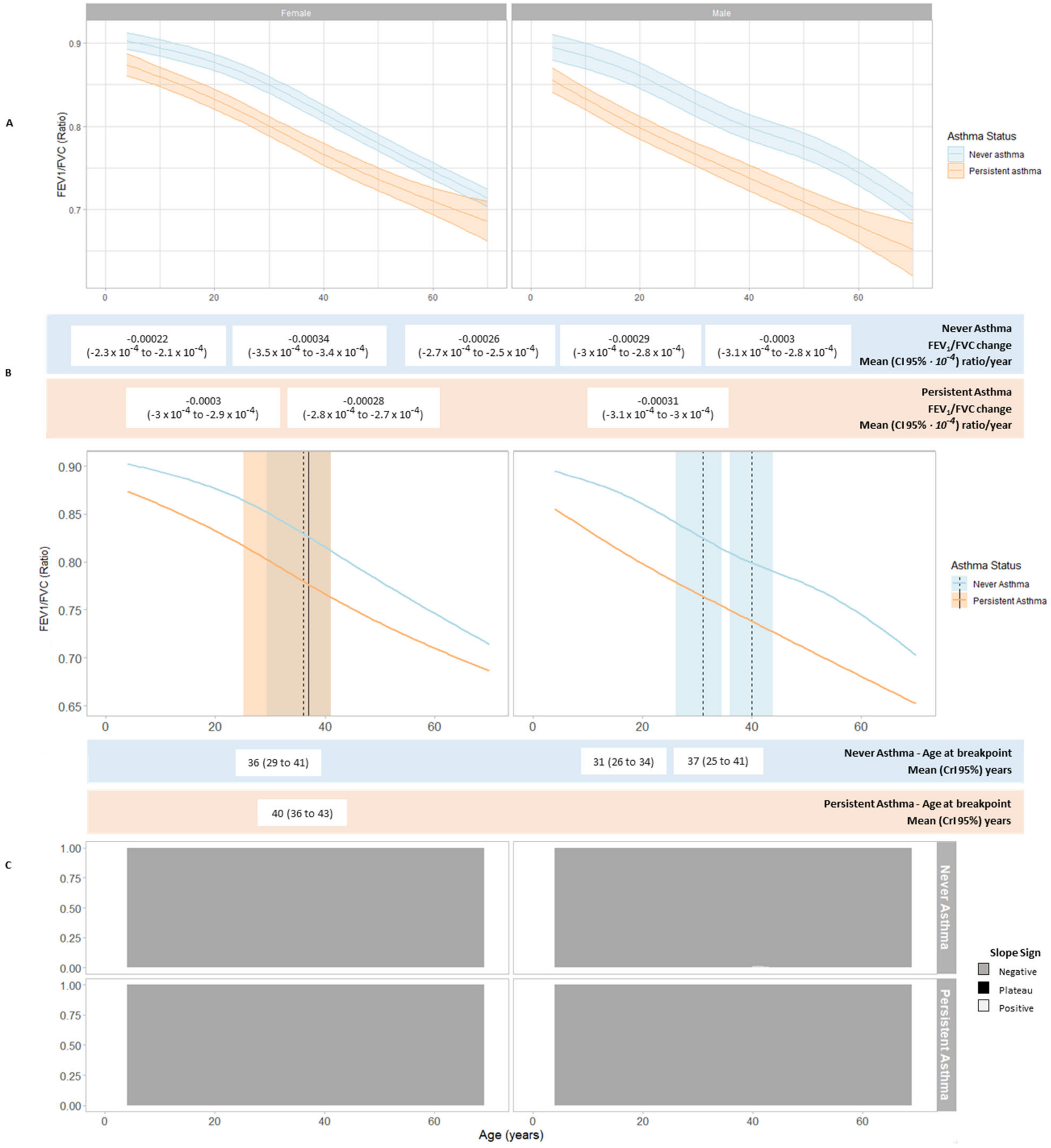

* Female: Never asthma – 11,025 (82·0%, 26,928 observations), Persistent asthma – 992 (7·4%, 2,386 observations); Male: Never asthma – 10,230 (83·6%, 24,779 observations), Persistent asthma – 984 (8·0%, 2,345 observations)

(b)

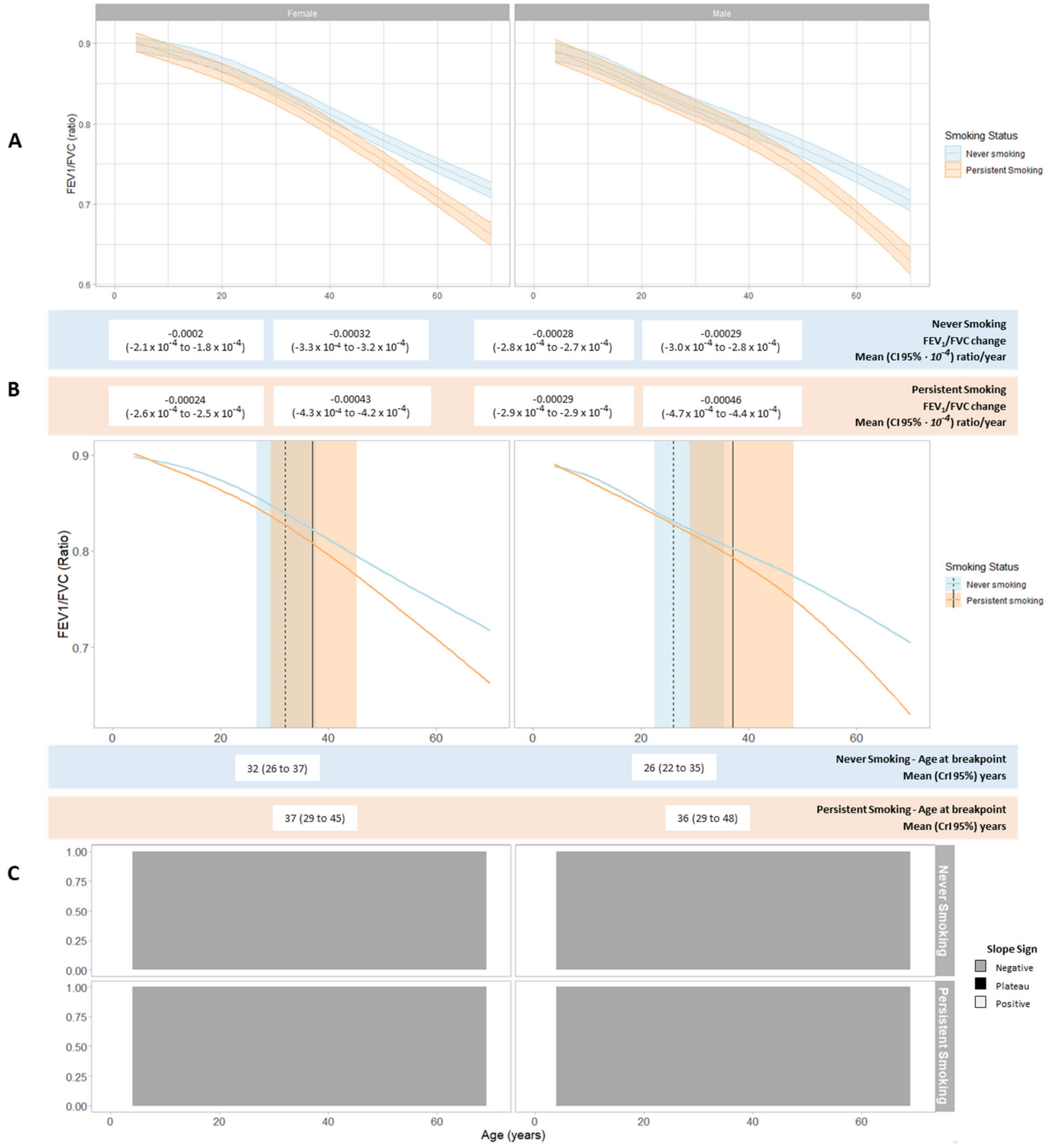

† Female: Never smoking – 7,590 (56·5%, 18,541 observations), Persistent smoking – 2,039 (15·2%, 4,729 observations); Male: Never smoking – 6,124 (50·1%, 14,799 observations), Persistent smoking – 2,082 (17·0%, 4,801 observations)

SUPPLEMENTARY REFERENCES

1 Boyd A, Golding J, Macleod J, et al. Cohort Profile: the 'children of the 90s'--the index offspring of the Avon Longitudinal Study of Parents and Children. *Int J Epidemiol* 2013; **42**:111-27.

2 Fraser A, Macdonald-Wallis C, Tilling K, et al. Cohort Profile: the Avon Longitudinal Study of Parents and Children: ALSPAC mothers cohort. *Int J Epidemiol* 2013; **42**:97-110.

3 Northstone K, Lewcock M, Groom A, et al. The Avon Longitudinal Study of Parents and Children (ALSPAC): an updated on the enrolled sample of index children in 2019. *Wellcome Open research* 2019; **4**:51

4 Harris PA, Taylor R, Thielke R, et al. Research electronic data capture (REDCap) – A metadata-driven methodology and workflow process for providing translational research informatics support. *J Biomed Inform* 2009; **42**:377-81.

5 Wang G, Hallberg J, Bergström PU, et al. Assessment of chronic bronchitis and risk factors in young adults: results from BAMSE. *Eur Respir J* 2021; **57**:2002120.

6 Wang G, Hallberg J, Faner R, et al. Plasticity of Individual Lung Function States from Childhood to Adulthood. *Am J Respir Crit Care Med* 2023; **207**:406-415.

7 Wickman M, Kull I, Pershagen G, Nordvall SL. The BAMSE project: presentation of a prospective longitudinal birth cohort study. *Pediatr Allergy Immunol* 2002; **Suppl 15**:11-13.

8 Janson C, Anto J, Burney P, et al. The European Community Respiratory Health Survey: what are the main results so far? *Eur Respir J* 2001; **18**:598–611.

9 Guxens M, Ballester F, Espada M, et al. Cohort profile: the INMA-INfancia y Medio Ambiente-(Environment and Childhood) Project. *Int J Epidemiol* 2012; **41**:930–940.

10 Wijga AH, Kerkhof M, Gehring U, et al. Cohort profile: the Prevention and Incidence of Asthma and Mite Allergy (PIAMA) birth cohort. *Int J Epidemiol* 2014; **43**:527–535.

11 Ackermann-Liebrich U, Kuna-Dibbert B, Probst-Hensch NM, et al. Follow-up of the Swiss cohort study on Air pollution and lung diseases in adults (SAPALDIA 2) 1991-2003: methods and characterization of participants. *Soz Praventivmed* 2005; **50**:245-263.

12 Matheson MC, Abramson MJ, Allen K, et al. Cohort Profile: The Tasmanian Longitudinal Health STUDY (TAHS). *Int J Epidemiol* 2017; **46**:407-408i.

13 Hospers JJ, Schouten JP, Weiss ST, Postma DS, Rijcken B. Eosinophilia is associated with increased all-cause mortality after a follow-up of 30 years in a general population sample. *Epidemiology* 2000; **11**:261–268.

14 van der Lende R, Kok T, Peset R, Quanjer PH, Schouten JP, Orie NG. Longterm exposure to air pollution and decline in VC and FEV1. Recent results from a longitudinal epidemiologic study in the Netherlands. *Chest* 1981; **80(1 Suppl)**:23-6.

15 Benet M, Albang R, Pinart M, et al. Integrating Clinical and Epidemiologic Data on Allergic Diseases Across Birth Cohorts: A Harmonization Study in the Mechanisms of the Development of Allergy Project. *Am J Epidemiol* 2019; **188**:408-417.

16 Hohmann C, Pinart M, Tischer C, et al. The development of the MeDALL Core Questionnaires for a harmonized follow-up assessment of eleven European birth cohorts on asthma and allergies. *Int Arch Allergy Immunol* 2014; **163**:215-24.

17 WHO Multicentre Growth Reference Study Group. WHO Child Growth Standards based on length/height, weight and age. *Acta Paediatr Suppl* 2006; **450**:76-85.

18 de Onis M, Onyango AW, Borghi E, Siyam A, Nishida C, Siekmann J. Development of a WHO growth reference for school-aged children and adolescents. *Bull World Health Organ* 2007; **85**:660-7.

19 Must A, Anderson SE. Body mass index in children and adolescents: considerations for population-based Applications. *Int J Obes* 2006; **30**:590-4.

20 World Health Organization. Obesity: preventing and managing the global epidemic. Report of a WHO consultation. *World Health Organ Tech Rep Ser* 2000; **894**:1–253.

21 van Buuren S, Groothuis-Oudshoorn K. mice: Multivariate Imputation by Chained Equations in R. *J Stat Soft* 2011; **45**:1-67.

22 Fong C, Ratkovic M, Imai K (2022). CBPS: Covariate Balancing Propensity Score. R package version 0.23. Available from https://CRAN.R-project.org/package=CBPS (last accessed May 2, 2024)
